# Ex Vivo Quantification of Calcification Stiffness in Aortic Stenosis: Biomechanical Data from Resected Human Valves

**DOI:** 10.1007/s10439-025-03869-x

**Published:** 2025-10-09

**Authors:** Takashi Shirakawa, Kazuo Shimamura, Koichi Maeda, Shin Yajima, Ai Kawamura, Takuji Kawamura, Daisuke Yoshioka, Shigeru Miyagawa

**Affiliations:** 1 Department of Cardiovascular Surgery, Graduate School of Medicine, The University of Osaka, Yamadaoka 2-2, Suita, Osaka 565-0871 Japan; 2 Department of Cardiovascular Surgical Technology Innovation, Graduate School of Medicine, The University of Osaka, Osaka, Japan

**Keywords:** Aortic stenosis, Calcification, Mechanical stiffness, Quantitative data, Compression test

## Abstract

**Purpose:**

Aortic stenosis (AS) is characterized by progressive calcification of the aortic valve. While imaging can assess the extent and localization of calcification, intraoperative findings suggest substantial variability in mechanical stiffness. Quantitative biomechanical evaluation is needed to inform optimized treatment strategies. We aimed to quantify the mechanical stiffness of calcified nodules in human AS.

**Methods:**

We performed ex vivo compression testing on 129 calcified nodules resected from 46 patients undergoing surgical aortic valve replacement for severe AS. Stress–strain relationships were measured to characterize the mechanical behavior of the nodules, and two stiffness parameters—compression strength (CS) and compression energy (CE)—were defined. These parameters were compared with the computed tomography (CT) density of the region from which each nodule was resected.

**Results:**

Calcified nodules exhibited wide variation in reactive stress, with maximum values in low strain regions ranging from 60 to 100-fold higher than the minimum. The stress–strain curves demonstrated three-phase pattern consisting of an initial increase, plateau phase, and steep rise in stress. The median CS increased from 0.38 MPa at 10% strain to 1.73 MPa at 50% strain, and median CE from 0.020 to 0.43 J/cm^3^ across the same range. The Pearson’s correlation coefficients between CT density and these parameters ranged from 0.291 to 0.454.

**Conclusion:**

Some nodules demonstrated marked reactive stress even at low strain levels, indicating strong resistance to compression with minimal deformation. This study provides reference data on the biomechanical stiffness of calcification in human AS.

**Supplementary Information:**

The online version contains supplementary material available at 10.1007/s10439-025-03869-x.

## Introduction

Aortic valve stenosis (AS) is one of the most common valvular heart diseases requiring surgical intervention in its advanced stages [1–3]. In recent years, the treatment strategy has shifted dynamically from surgical aortic valve replacement (SAVR) to transcatheter aortic valve replacement (TAVR) [4, 5]. In SAVR, the calcified leaflets are surgically resected, whereas in TAVR, they are compressed against the aortic annulus and Valsalva’s sinus. This difference makes TAVR more susceptible to complications associated with calcification, such as suboptimal valve expansion, paravalvular leakage, aortic root rupture, or stroke [6–9].

Previous studies have indicated that calcium density and burden in computed tomography (CT) images are important factors in the severity and treatment of AS [10–13]. The CT-based calcium score is calculated as a weighted sum of calcium density and is widely used in clinical practice. However, such calcium scores do not directly reflect the actual mechanical stiffness of the lesions. Based on observations during SAVR, calcified nodules on AS valves vary not only in size and shape but also particularly in stiffness. Some nodules are fragile and easily crushed, while others are rigid and require specialized surgical instruments to remove. In contrast, calcifications cannot be excised during TAVR, and numerical simulations have demonstrated that high stress tends to concentrate around calcified regions during the procedure [14, 15]. In the future, if the mechanical properties of calcific lesions can be estimated from imaging and clinical data, the accuracy of simulations could be improved on a patient-specific basis. Alternatively, statistical analysis comparing preoperative stiffness estimates with postoperative outcomes could facilitate risk stratification and optimization of treatment strategies in clinical practice. To enable such approaches, it is first essential to establish direct measurements of calcified nodules in real specimens from patients.

We investigated the mechanical response of calcified nodules in AS valves by ex vivo compression tests using load testing machines. We also defined and calculated two stiffness parameters—Compression Strength and Energy—for the calcified nodules and compared them with the CT numbers of the corresponding lesions. This study aims to provide reference data and quantitative definition of stiffness for AS calcification as foundational metrics for future research.

## Materials and Methods

### Study Population

Patients who underwent SAVR for severe aortic stenosis at one of the four participating institutions—Kansai Rosai Hospital (August 2017–February 2018), Kinan Hospital (April 2018–October 2018), Suita Tokushukai Hospital (January 2022–December 2022), and Osaka University Hospital (January 2022–December 2022)—were consecutively screened and enrolled in this study. Inclusion was limited to patients who had preoperative electrocardiogram-gated CT imaging that included the aortic valve and underwent SAVR as part of standard treatment. As native aortic valves are explanted during prosthetic valve replacement, these surplus specimens were used for experimental analysis.

From the preoperative clinical status, we selected variables potentially related to calcification stiffness. Symptoms were recorded, and cardiac function was graded according to the New York Heart Association (NYHA) classification. Among preoperative echocardiographic parameters, we included indices reflecting AS severity—peak velocity, mean pressure gradient, and aortic valve area index (AVAi)—as well as left ventricular ejection fraction (LVEF). Patient history was reviewed for comorbidities including hypertension, diabetes mellitus, hemodialysis, coronary artery disease, and the presence of a bicuspid aortic valve. No restrictions were placed on clinical background or imaging findings.

The study was conducted in accordance with the principles outlined in the Declaration of Helsinki. Ethical approval was obtained from the Institutional Review Board of Kansai Rosai Hospital (Approval No. 17C055g). All participants provided written informed consent prior to enrollment in the study.

### Ex Vivo Load–Compression Test

**Fig. 1:**
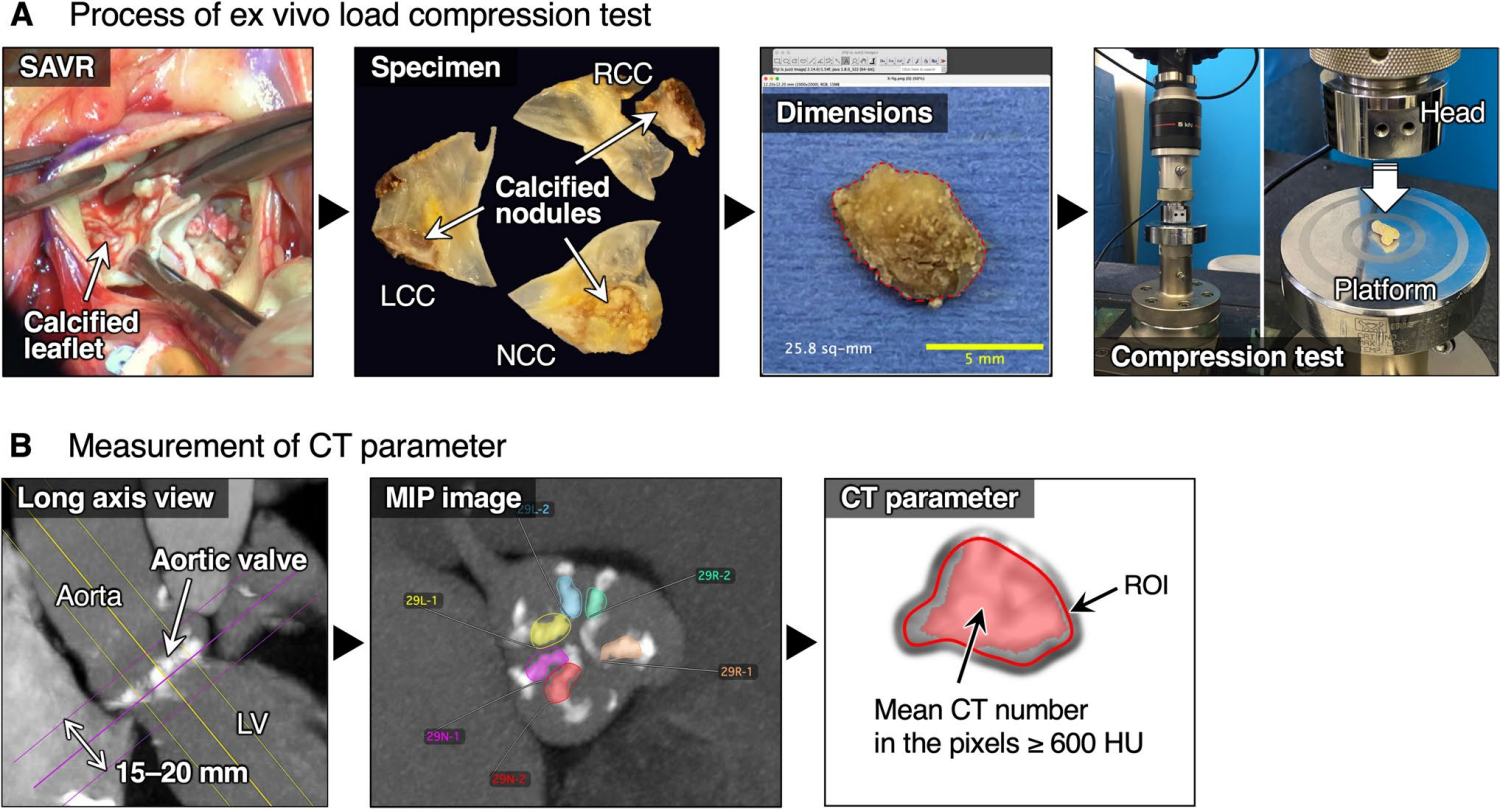
Ex vivo load compression test and CT density. **A** Calcified aortic valve leaflets were resected during SAVR. Intact calcified nodules without visible damage were isolated from the resected valves. Nodule height was measured using calipers, and projected area was determined from frontal images using image analysis software. Each calcified nodule was subjected to compression testing using a load testing machine. **B** A 15–20-mm-thick slab parallel to the virtual basal ring was used to encompass all valvular calcifications. ROIs were defined on the generated MIP image to enclose the corresponding calcified nodules. The mean CT number was calculated in high-density pixels with CT values ≥ 600 HU within each ROI

Fig. 1A shows the process of a load compression test. Calcified AS valves were resected during surgery and preserved in 10% neutral buffered formalin at room temperature until mechanical testing. In each resected valve, nodules larger than 2 mm that retained their shape were selected for analysis. The height of each nodule was measured using calipers, and the projected area was determined from a frontal view using Fiji (an open-source distribution of ImageJ/ImageJ2, developed by the Fiji community, version 2.14.0/1.54f) [16].

Load-compression tests were performed using testing machines: INSTRON 5982 (Instron Co., Ltd., MA, USA) at the Hyogo Prefectural Institute of Technology and INSTRON 5569 at the Industrial Technology Center of Wakayama Prefecture. A single calcified nodule was placed on the machine’s specimen platform with its basal area (left ventricular side) facing downward. The applied load (unit: N, Newton) and compression distance (mm) were recorded every 20 milliseconds at a compression speed of 2.00 mm/min until reaching a load limit of 2000 N (203.9 kg). The compression distance origin was defined when the preload surpassed 0.02 N/mm^2^ (2.04 g/mm^2^). The measurement accuracy of the machines was ± 0.1 N (10.2 g) for load and ± 0.01 mm for distance.

### CT Imaging of Calcified Nodules

Fig. 1B shows the measurement of the mean CT number (CT attenuation value, calcium density in CT images) of a nodule. A maximum intensity projection (MIP) CT image with a thickness of 15–20 mm was generated through a frontal view of the virtual basal ring to encompass all valvular calcifications. Regions of interest (ROI) were subsequently defined in the MIP image, enclosing the corresponding calcified nodules. High-density pixels with a CT number of ≥ 600 HU were selected to calculate the mean CT number within the ROI of each nodule using OsiriX MD version 14.1 (Pixmeo SARL, Geneva, Switzerland).

### Stress–Strain Relationship under Compression

The stress–strain relationship for each nodule was obtained by calculating stress at every 0.05 strain increment based on the applied load and measured displacement. In engineering terms, stress is defined as the loading force per unit area (force divided by the loading area), and strain is defined as the rate of change in displacement (displacement divided by the initial length). However, because the calcified nodules deform in size and shape significantly during the compression process, representative values were calculated as follows:

The initial volume, ${V}_{0}$ (unit: mm^3^), was assumed to be a fixed value obtained by multiplying the initial projected area, ${A}_{0}$ (mm^2^), by the height, ${H}_{0}$ (mm):

$${V}_{0}={A}_{0}\bullet {H}_{0}$$

The contact ratio with the testing machine’s head and platform, ${R}_{c}$, was assumed to 0.5 at the start of compression and was linearly increased to 1.0 when the sample was compressed to half of its original height. The stress $\sigma$ (MPa) was obtained by dividing the compressive force $F$ (N) by the loading area $A$ (mm^2^):

$$\sigma =F/A , where A={R}_{c}\left({V}_{0}/H\right)$$

The strain $\varepsilon$ was obtained by dividing the displacement $L$ (mm) by the initial height ${H}_{0}$:

$$\varepsilon =L/{H}_{0}$$

### Definition of Stiffness Parameters

**Fig. 2:**
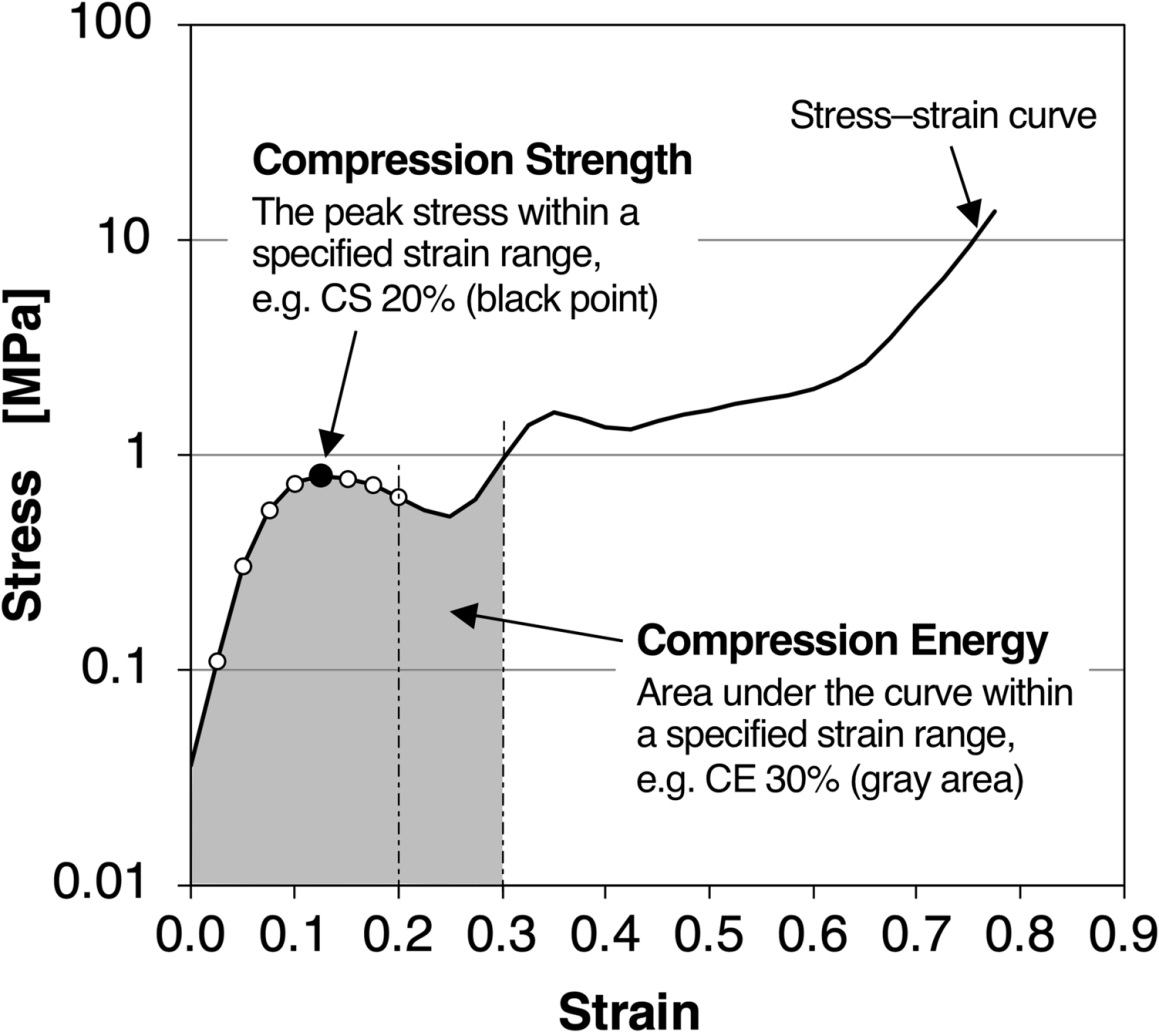
Definition of compression strength and compression energy. A representative illustration of compression strength and compression energy, as defined in this study

To quantify the mechanical stiffness of calcified nodules from the stress–strain relationship described above, we defined two parameters—Compression Strength and Compression Energy—at specific strain levels as follows:

1. Compression Strength (CS, unit: MPa): The peak stress observed during the compression process up to the specified strain level. For example, CS20 represents the maximum stress between 0 and 20% strain (black point in Fig. 2).
2. Compression Energy (CE, unit: J/cm^3^): The energy required to compress a nodule to a given strain level. This was calculated as the integral of the stress–strain curve, representing the area under the curve. For example, CE30 corresponds to the area between 0 and 30% strain (gray area in Fig. 2).

While the stress–strain curve reflects the raw mechanical behavior during compression, CS reflects the amount of stress the nodule can exert on surrounding tissues, whereas CE indicates the amount of work a compressing object, such as an endovascular balloon, must perform to achieve deformation.

### Statistical Analysis

Given the variability in stiffness values, all data points were retained, and no outliers were excluded. The stress–strain relationship for each calcified nodule was obtained at every 0.05 strain increment. Based on the data across all nodules, a distribution curve of stress versus strain was generated to illustrate the possible range of stress values at each strain level. Meanwhile, the CS and CE were calculated for each nodule at every 0.1 strain increment, and their distributions were presented as quartile ranges. In addition, we assessed the Pearson’s correlation coefficients between those stiffness values and the mean CT numbers (calcium density in CT images) of calcified nodules. Statistical analyses were performed using R version 4.4.2 (R Foundation for Statistical Computing, Vienna, Austria). Data are presented as *n* (%) for categorical variables and median [25th, 75th percentiles] for continuous variables.

## Results

### Study Population and Calcified Nodules

**Fig. 3:**
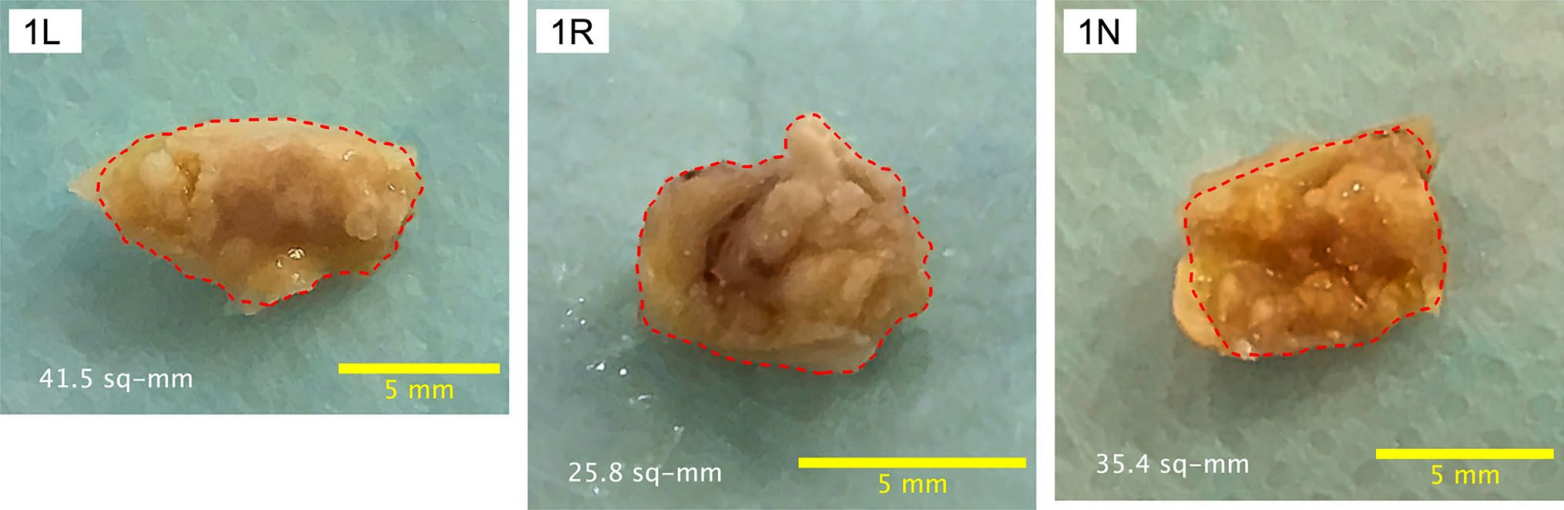
Calcified nodules resected from an AS valve. Calcified nodules resected from an AS valve of Case 1. The top-left legend in a white box indicates the case number and the cusp from which each nodule was derived. L, R, and N refer to the left, right, and non-coronary cusps, respectively. The lower legends indicate the outlined nodule area (red dashed border), excluding any surrounding soft tissue or superficial attachments, and the scale bar

A total of 46 patients (median age 75.5 years; 20 males) were enrolled, including 25 who underwent isolated SAVR (Table 1). Among the study cohort, 34 patients (73.9%) had heart failure classified as NYHA class II or higher, 30 (65.2%) had hypertension, 12 (26.1%) had diabetes mellitus, and 27 (58.7%) had a history of coronary artery disease or significant stenosis (> 50%) on preoperative CT. Eight patients (17.4%) were receiving hemodialysis, and 6 (13.0%) had a bicuspid aortic valve. Preoperative echocardiographic findings showed a peak velocity of 4.7 [4.1, 5.1] m/s, AVAi of 0.49 [0.41, 0.55] cm^2^/m^2^, and LVEF of 67.5 [63.0, 74.8] %. From the AS valves, a total of 129 calcified nodules were obtained for mechanical analysis (Table 2). Figure 3 shows representative images of nodules from one case. Images of all nodules are provided in Supplemental Fig. 1.

**Table 1:** Patient characteristics

Patient characteristics	
Number of patients	46
Age	75.5 [71.3, 78.8]
Sex (male)	20 (43.5)
Body-surface area [m^2^]	1.51 [1.41, 1.66]
NYHA class	
I	12 (26.1)
II	23 (50.0)
III	7 (15.2)
IV	4 (8.7)
Symptom	
None	12 (26.1)
Heart failure	27 (58.7)
Syncope	7 (15.2)
Chronic status	
Hypertension	30 (65.2)
Diabetes	12 (26.1)
Hemodialysis	8 (17.4)
Coronary artery disease	27 (58.7)
Bicuspid aortic valve	6 (13.0)
Echocardiography	
Peak velocity [m/s]	4.7 [4.1, 5.1]
Mean pressure gradient [mmHg]	54 [40, 64]
AVAi [cm^2^/m^2^]	0.49 [0.41, 0.55]
LVEF [%]	67.5 [63.0, 74.8]
Surgery	
Isolated AVR	25 (54.3)
Concomitant CABG	9 (19.6)

*AVAi* indexed aortic valve area, *AVR* aortic valve replacement, *CABG* coronary artery bypass grafting, *LVEF* left ventricular ejection fraction, *NYHA* New York Heart Association

Data are presented as *n* (%) for categorical variables and median [25th, 75th percentiles] for continuous variables.

**Table 2:** Clinical background of resected calcium nodules

Background of resected calcium nodules	
Number of calcium nodules	129
Age	76.0 [72.0, 79.0]
Sex (male)	63 (48.8)
Peak velocity [m/s]	4.7 [4.3, 5.1]
AVAi [cm^2^/m^2^]	0.48 [0.40, 0.53]
LVEF [%]	69.0 [61.0, 75.0]
Hypertension	84 (65.1)
Diabetes	27 (20.9)
Hemodialysis	27 (20.9)
Coronary artery disease	83 (64.3)
Bicuspid aortic valve	13 (10.1)
Mean CT number in CT images [HU]	937 [842, 1018]
Calcium area in CT images [mm^2^]	23.67 [15.9, 35.22]
Collection to experiment [days]	9 [3, 13]

Data are presented as *n* (%) for categorical variables and median [25th, 75th percentiles] for continuous variables

### Stress–Strain Relationship

**Fig. 4:**
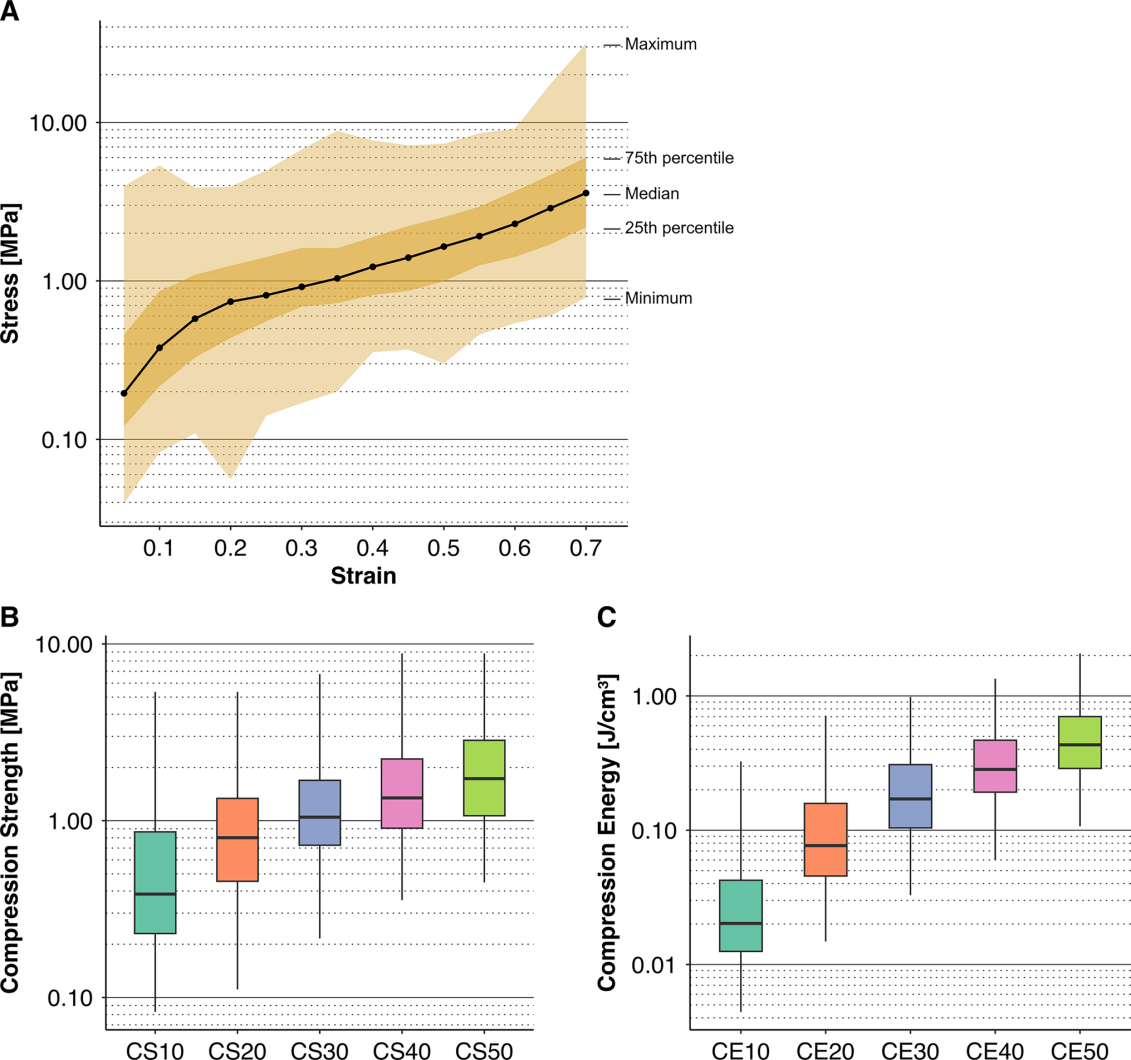
Stress–strain relationships. **A** Quartile ranges of stress during compression testing for all 129 nodules are shown. This figure corresponds to an overlay of the stress–strain curves of all specimens, representing the possible range of stress values at each strain level. Data were processed at every 0.05 strain increment. **B**, **C** Boxplots display the quartile ranges for each of the five Compression Strength (CS) and Compression Energy (CE) parameters as defined in the Methods. CEx, Compression Energy at x% strain; CSy, Compression Strength at y% strain

The stress–strain relationship was presented as quartile distributions of stress at every 0.05 strain increment across all 129 calcified nodules (Table 3). Figure 4A illustrates this trend on a logarithmic scale. The median stress was 0.378 MPa (38.5 g/mm^2^) at a strain of 0.1, remained below 1 MPa (102 g/mm^2^) until a strain around 0.35, and increased to 1.65 MPa (168 g/mm^2^) at 0.5, reaching 3.59 MPa (366 g/mm^2^) at a strain of 0.7. The progression of stress rise, as seen in Figure 4A, showed a rapid increase up to approximately 0.15 strain, a plateau-like phase between 0.15 and 0.4, followed by another phase of steep rise beyond 0.4 strain (note that the stress axis is logarithmic). Stress–strain curves of individual nodules are shown in Supplemental Fig. 2.

**Table 3:** Stress distribution across calcified nodules

	Strain													
	0.05	0.10	0.15	0.20	0.25	0.30	0.35	0.40	0.45	0.50	0.55	0.60	0.65	0.70
Maximum	3.960	5.356	3.869	3.927	4.969	6.750	8.837	7.692	7.178	7.325	8.550	9.152	17.49	31.49
75th percentile	0.458	0.859	1.094	1.248	1.413	1.616	1.608	1.890	2.221	2.523	2.942	3.685	4.662	6.029
Median	**0.195**	**0.378**	**0.578**	**0.740**	**0.812**	**0.918**	**1.037**	**1.227**	**1.403**	**1.647**	**1.916**	**2.292**	**2.878**	**3.585**
25th percentile	0.121	0.217	0.329	0.436	0.556	0.687	0.724	0.815	0.865	0.996	1.254	1.418	1.700	2.183
Minimum	0.040	0.083	0.110	0.056	0.141	0.170	0.201	0.355	0.369	0.302	0.459	0.542	0.604	0.785
75th percentile/25th percentile	3.79	3.96	3.33	2.86	2.54	2.35	2.22	2.32	2.57	2.53	2.35	2.60	2.74	2.76
Maximum/minimum	100.0	64.6	35.3	70.2	35.2	39.8	44.1	21.7	19.4	24.3	18.6	16.9	29.0	40.1

The stress–strain relationship as quartile distributions of stress at every 0.05 strain increment across all 129 calcified nodules

In the lower strain range, the 75th percentile was approximately four times the 25th percentile, while in the higher strain range, this ratio decreased to around 2.5. In contrast, the maximum value in the lower strain range reached 60–100 times the minimum value, whereas in the higher strain range, this ratio rapidly decreased to between 15 and 45. Taking into account the logarithmic scale, Figure 4A indicates that the upper 25% of calcified nodules exhibited a markedly broader distribution of reactive stress compared to the other quartile ranges of the remaining nodules.

### Compression Strength and Compression Energy

The median and IQR of CS for strain were as follows: 0.38 [0.23, 0.86] MPa at 10% strain, 0.80 [0.45, 1.34] MPa at 20%, 1.05 [0.73, 1.69] MPa at 30%, 1.35 [0.91, 2.23] MPa at 40%, and 1.73 [1.07, 2.85] MPa at 50%. Similarly, the median and IQR of CE were 0.020 [0.013, 0.042] J/cm^3^ at 10% strain, 0.077 [0.046, 0.158] J/cm^3^ at 20%, 0.17 [0.10, 0.31] J/cm^3^ at 30%, 0.28 [0.19, 0.47] J/cm^3^ at 40%, and 0.43 [0.29, 0.70] J/cm^3^ at 50%. Figures 4B and C presents boxplots illustrating these quartile distributions for CS and CE, respectively.

### Mechanical Stiffness and CT Density

**Fig. 5:**
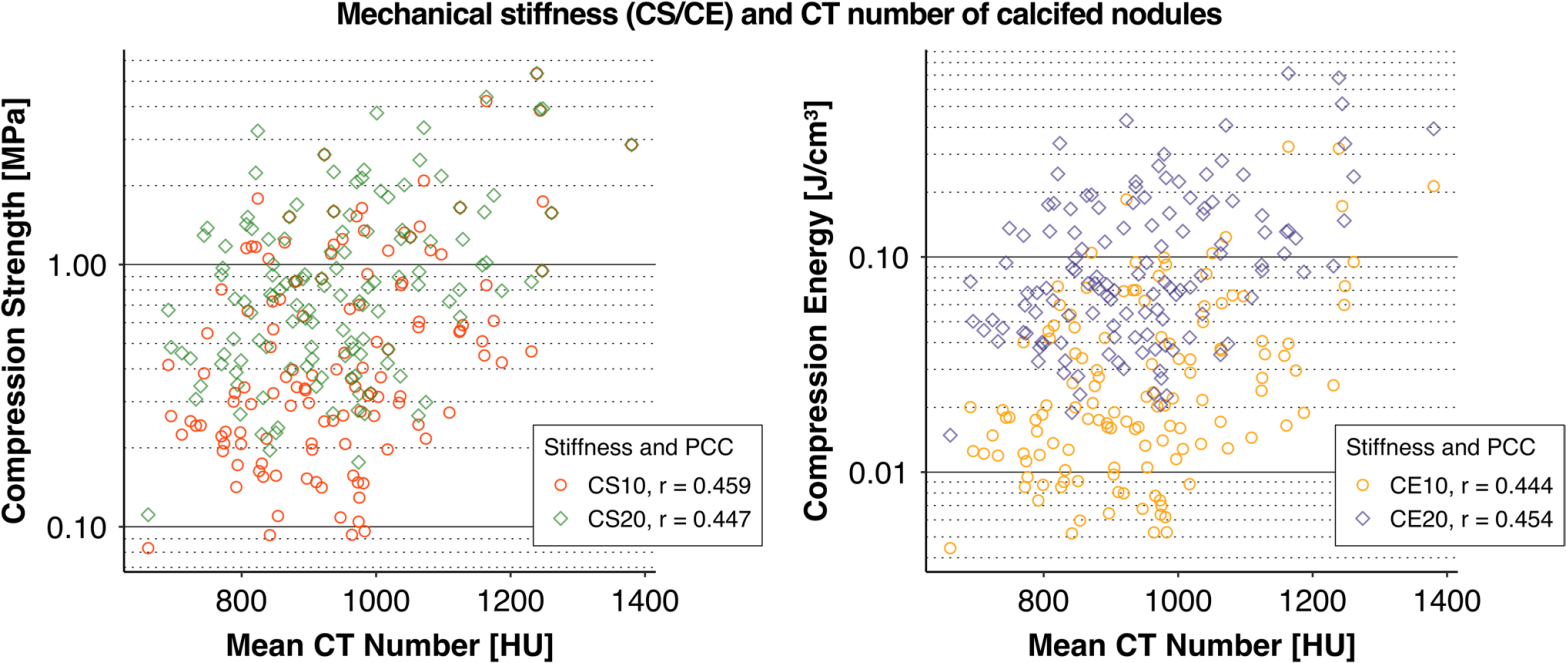
CT density versus CS and CE. Plots of CS and CE values at 10 and 20% strain versus the CT numbers of calcified nodules are shown. The Pearson’s correlation coefficients were approximately 0.45 at these strain levels. Lower correlation values were observed at 30, 40, and 50% strain. The correlation coefficients were statistically significant, with *p* < 0.001. CEx compression energy at x% strain, CSy compression strength at y% strain, PCC Pearson’s correlation coefficient

The Pearson’s correlation coefficients between the mean CT numbers of calcified nodules and CS were 0.459 for CS10, 0.447 for CS20, 0.393 for CS30, 0.348 for CS40, and 0.291 for CS50. For CE, the coefficients were 0.444 for CE10, 0.454 for CE20, 0.441 for CE30, 0.399 for CE40, and 0.340 for CE50. The numbers following CS and CE indicate the strain level as a percentage. All correlation coefficients were statistically significant, with *p* < 0.001. Figure 5 shows representative plots of CS and CE values at 10 and 20% strain versus the mean CT numbers. Clinical parameters and corresponding CS and CE are provided in Supplemental Data 1.

## Discussion

AS is caused by progressive leaflet stiffening from calcific lesions, leading to restricted valve opening and reduced cardiac output. SAVR allows direct removal of even extremely rigid calcifications, whereas TAVR relies solely on compressing them against surrounding tissues. Large, rigid nodules can therefore increase the risk of annular rupture, incomplete valve expansion, or paravalvular regurgitation—events linked to worse long-term outcomes. Originally introduced for high-risk elderly patients, TAVR is now being offered to younger, lower-risk populations. In these patients, the priority shifts from procedural minimal invasiveness to ensuring that the prosthetic valve will function reliably over the much longer remaining lifespan. Accurate preoperative assessment of anatomical suitability is thus essential. While CT can assess calcification size and distribution, quantitative data on mechanical stiffness have been lacking.

The significance of this study lies in its experimental and quantitative evaluation of this clinically relevant problem using a mechanical testing approach. Furthermore, by defining and applying the concepts of CS and CE, this work establishes a foundation for future biomechanical and imaging-based studies of aortic valve calcification.

### Biomechanical Insights into the Compression of Calcified Nodules

The stress distribution shown in Fig. 4A indicates that while stress values for the lower 75% of calcified nodules clustered within a relatively narrow range, the upper 25% exhibited wide dispersion. This implies that surgeons have likely encountered, perhaps unknowingly, extremely stiff calcified nodules that exert substantial reactive force on surrounding tissues with only minimal deformation.

As described in the Methods, CS reflects the amount of force per unit area that a nodule exerts on surrounding tissues, whereas CE represents the amount of work required by a compressing device to achieve sufficient deformation of a calcified nodule. Importantly, CS and CE are not independent of each other. A nodule with high CS may resist deformation to the extent that a device attempting to achieve a high CE could cause fracture or even perforation—an event not unfamiliar to those with clinical experience in TAVR. From a surgical perspective, this phenomenon is readily understandable.

Conversely, high CE values can also result from nodules with low CS and large deformation capacity. In such cases, the stress–strain curve exhibits a horizontally stretched profile—an indication of a soft but extensively deformable lesion. This pattern is likewise consistent with clinical impressions of soft calcifications.

From a biological standpoint, calcific lesion formation is a multifactorial, multistage process involving endothelial injury, immune cell infiltration, osteoblastic differentiation, and mechanical stress [17, 18]. At the ultrastructural level, calcific deposits consist of densely packed calcium phosphate crystals forming a porous structure, interspersed with irregularly distributed organic material [19]. Progression from microcalcification to large deposits, along with changes in crystal composition and organic matrix content such as collagen, can alter lesion stiffness. Conversely, macroscopic stiffness values may provide indirect insight into the biological stage and tissue composition. The moderate correlations observed with CT numbers may reflect the difficulty in capturing ultrastructural heterogeneity within calcific deposits, as well as the lack of consideration of the temporal stage of AS severity and the influence of formalin fixation.

Formalin fixation stiffens proteins such as collagen fibers, while mineral components such as calcification are considered less affected [20–22]. Conversely, in bone, prolonged formalin fixation has been reported to cause calcium leaching [23]. Therefore, the proportion of organic elements in the calcified nodules, as well as the duration of fixation (median 9 days, maximum 61 days), may have influenced the mechanical properties observed in this study.

Although we calculated Young’s modulus from the stress–strain curve, the results were inconsistent, likely due to measurement noise at very small strains and the influence of collagen denaturation after formalin fixation. Therefore, this parameter is not included.

### Compression Phases of Calcified Nodules

According to fracture mechanics, the crushing of a solid is characterized by the discontinuous formation of internal cracks and the subsequent release of internal stress through localized deformation [24]. The representative stress–strain curve observed in this study (Fig. 2) demonstrates three distinct phases. Such three-phase compression behavior is well recognized in material foams, including both metal and polymeric foams [25, 26]. In these foams, the porous architecture is formed by the aggregation of microstructures of varying sizes, and this structural arrangement underlies their characteristic crushing response. Calcified nodules in AS, likewise composed of irregularly sized microstructural elements assembled into a porous morphology [19], show an analogous pattern of three-phase behavior.

Stage I (initial elastic deformation) is characterized by a smooth, rapid increase in applied load over a short compression distance (Fig. 2, strain 0.0–0.1). Stage II is marked by a reduced or even negative slope in the load–compression curve, often accompanied by jagged fluctuations. These features likely reflect repeated microfracture formation and local stress relief within the calcified nodule (Fig. 2, strain 0.1–around 0.5). Stage III shows a renewed and steeper increase in load, attributed to the densification of fragmented particles trapped between the platform and compression head until the mechanical limit is reached (Fig. 2, strain around 0.5–0.9).

Based on our tentative criteria—an area under the stress–strain curve ≥ 0.7 (calculated as the ratio to a rectangle with height from stress 0.02 N/mm^2^ to stress at strain 0.5 and width of strain 0–0.5) or a negative slope between strain 0.05 and 0.5—three-phase behavior was observed in 118 of 129 nodules, while the remaining 11 showed gradual increases (Supplemental Fig. 2). This variability likely reflects the heterogeneous composition and structural irregularities of calcified nodules, unlike the uniformity of artificial materials. Alternatively, non-calcified fibrotic tissue may have been interspersed within the nodules.

In a preliminary analysis of Table 2, nodules with a three-phase pattern included 16.9% (20/118) from dialysis patients, compared with 63.6% among non–three-phase nodules (7/11; *p* = 0.002, Fisher’s exact test). Non–three-phase nodules also tended to have a lower calcified-to-basal area ratio (0.70 vs. 0.79; *p* = 0.087, Mann–Whitney *U* test). Previous reports have indicated that in female, younger patients, or those with rheumatic AS, the contribution of calcification to leaflet restriction is relatively modest [27, 28]. Similarly, in dialysis patients, diffuse leaflet thickening and stiffening may reduce the mechanical impact of calcification in stress–strain curves.

### CT-Based Estimation of Mechanical Stiffness

It has been suggested that the mechanical characteristics of calcification can be stratified into several groups based on calcium density in CT images [29], and it is generally recognized that a denser appearance on CT corresponds to greater stiffness. However, in the present study, the correlation coefficients between the mean CT numbers of calcified nodules and their actual mechanical properties—CS and CE—were approximately 0.45 or lower (Fig. 5). Considering the dispersion of the plotted data points, CT numbers alone seem insufficient to achieve satisfactory predictive capability. However, as noted above, the effects of formalin fixation are considered limited but cannot be ignored. The results should be interpreted as potentially influenced by storage duration and preservation methods.

While CT numbers are undoubtedly one of the important factors in the preoperative prediction of CS and CE, the structural heterogeneity and complexity of calcified lesions suggest that advanced modeling techniques, such as neural network models incorporating imaging features, patient demographics (e.g., age, sex), and disease severity indices (e.g., echocardiographic findings), will be required to approximate nodule stiffness [30, 31]. In this study, we provided these clinical data for all specimens in Supplemental Data 1, which may facilitate the development of predictive models using multivariable analysis.

### Implications for TAVR Simulation and Planning

Several studies have demonstrated that the extent of aortic valve calcification correlates not only with disease severity and prognosis but also with clinical outcomes and TAVR-related complications, such as paravalvular regurgitation and device malposition [6–9].

Numerical simulations and stress mapping have been reported to model the mechanical environment during TAVR [14, 15, 32]. These models provide insight into stress distribution within the valve, annulus, and adjacent structures. High-rigidity nodules will resist deformation and maintain their structure during deployment. This can lead to localized stress concentration on the annulus or sinus, increasing the risk of annular rupture, restricted expansion, or residual regurgitation. A key limitation, however, is the reliance on assumed material properties of calcifications, reducing physiological accuracy. As shown in this study, most calcified nodules exhibited three-phase compression patterns: initially resisting deformation and generating high reactive stress, followed by a plateau phase in which further crushing required relatively little stress. While calcifications can be manually excised in SAVR, the stress concentration is critical in TAVR, which depends on compressing calcific lesions against surrounding tissues. Of particular importance is whether the plateau phase is reached before stress levels rise to the threshold of annular or sinus rupture, or whether device expansion must be deliberately limited for safety—resulting in incomplete expansion, paravalvular regurgitation, or device malposition.

If mechanical properties can be predicted from preoperative CT, this may enable more accurate risk stratification and individualized planning. Integrating imaging-based prediction with empirical data could be key to optimizing long-term outcomes of TAVR, particularly in younger or lower-risk patients.

## Conclusions

We conducted ex vivo compression testing on 129 calcified nodules resected from 46 patients with aortic stenosis. Two quantitative parameters, CS and CE, were defined to characterize the biomechanical stiffness of nodules across a range of strain levels. The stress–strain relationship revealed that some nodules exhibited marked stiffness even at low strain levels, indicating the potential for strong reactive force with minimal deformation. The mechanical stiffness values established in this study provide the first set of quantitatively measured data for AS-related calcifications under conditions of standard clinical tissue preservation. Further rigorous histological analysis is warranted to validate the physiological relevance of the mechanical properties described herein.

### Limitations

This study was limited to 46 patients and 129 calcified nodules. The nodules varied in size, shape, and anatomical location due to biological heterogeneity. In addition, preservation in 10% neutral buffered formalin, although standard for clinical specimens, may have altered some mechanical properties of the nodules. Mechanical testing was performed under uniaxial compression in a controlled in vitro setting, which does not fully replicate the complex, multidirectional forces acting on the valve during TAVR. Furthermore, although we analyzed nodules that were grossly intact and larger than 2 mm, smaller or more fragmented calcifications were excluded, which may limit generalizability.

## Supplementary Information

Below is the link to the electronic supplementary material.

Supplementary file1 (DOCX 73636 kb)

Supplementary file2 (XLSX 41 kb)

## Data Availability

Datasets generated and analyzed during the study are available as supplementary materials.
