# Supplementary material for: Ex Vivo Quantification of Calcification Stiffness in Aortic Stenosis: Biomechanical Data from Resected Human Valves
Source: Ann Biomed Eng. 2025 Oct 9;53(12):3389–99. doi: 10.1007/s10439-025-03869-x (PMC12685988; doi:10.1007/s10439-025-03869-x)
Supplement: Supplementary file 1 — Supplementary file1 (DOCX 73636 kb) [file 10439_2025_3869_MOESM1_ESM.docx]

**Supplemental Figure 1.** Calcified nodules resected from AS valves. The left-upper legend in a white box indicates the case number and cusp; for example, "3L" refers to the left coronary cusp of Case 3, matching those shown in Supplementary Figure 2. The lower legends indicate nodule area (red dashed border) and the scale bar.


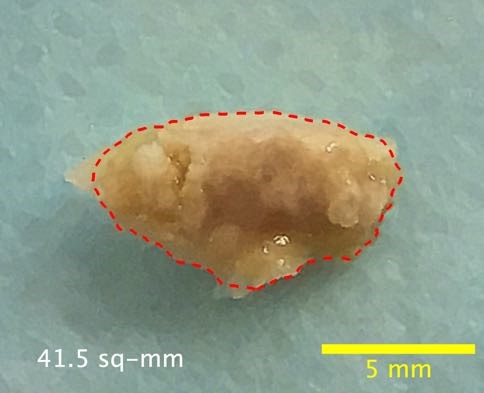


1

L


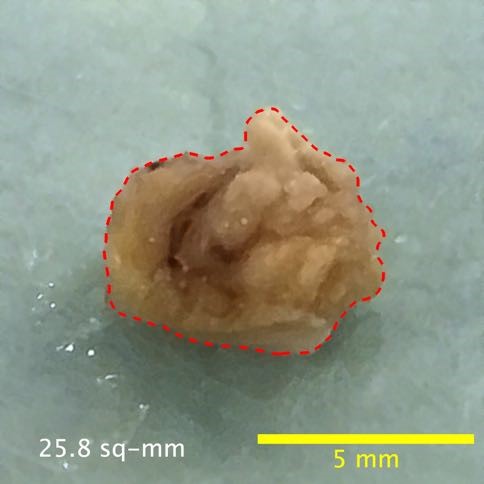


1

R


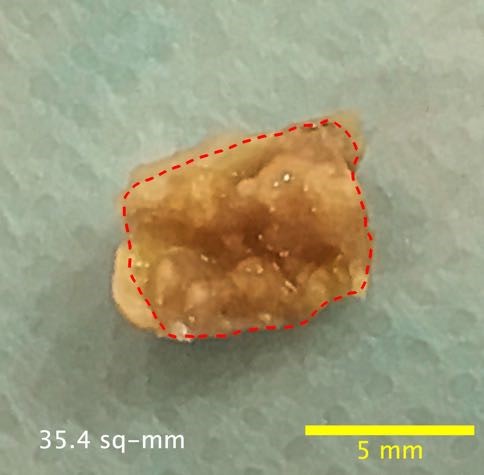


1

N


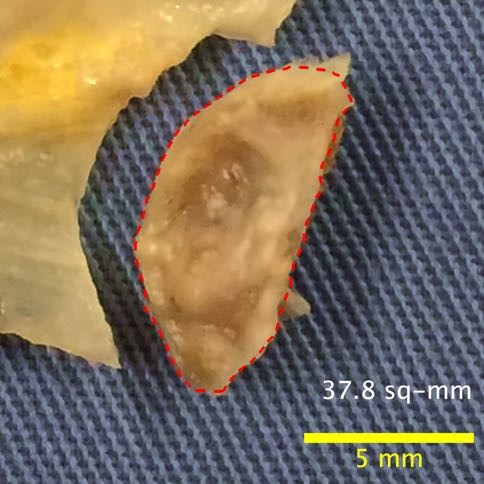


2

L

1


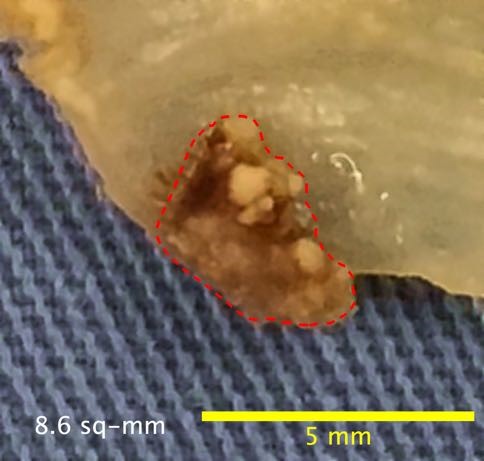


2

L

2


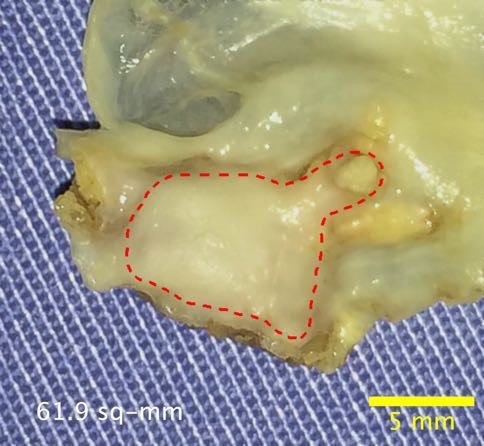


2

R


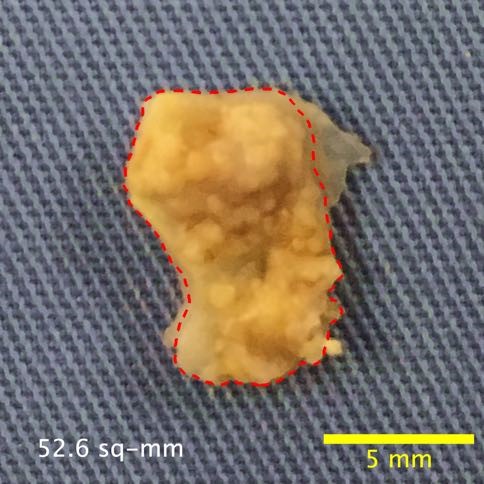


2

N


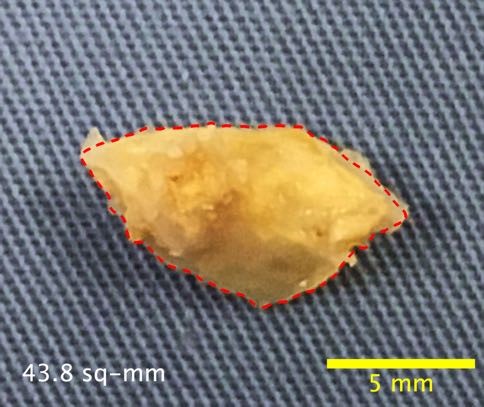


3

L


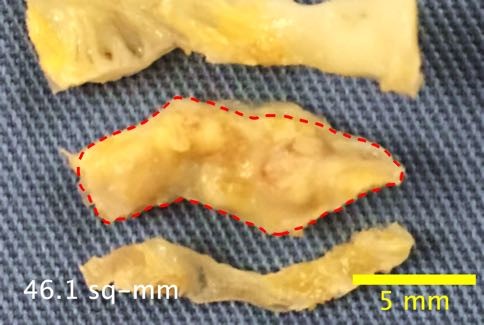


3

R


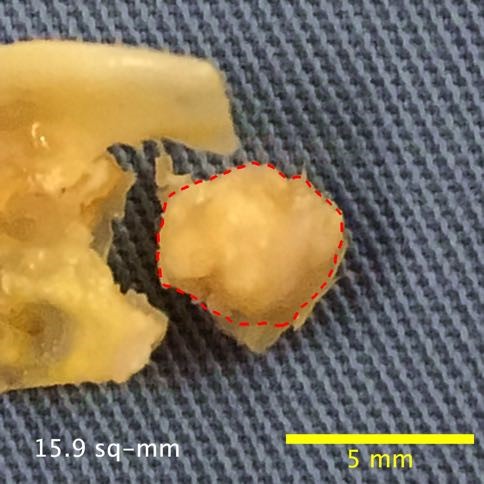


3

N

1


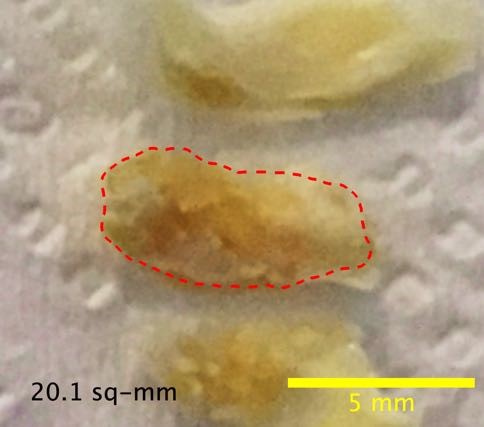


3

N

2


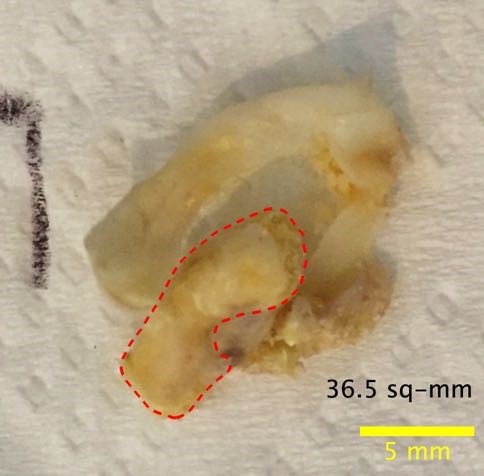


4

L


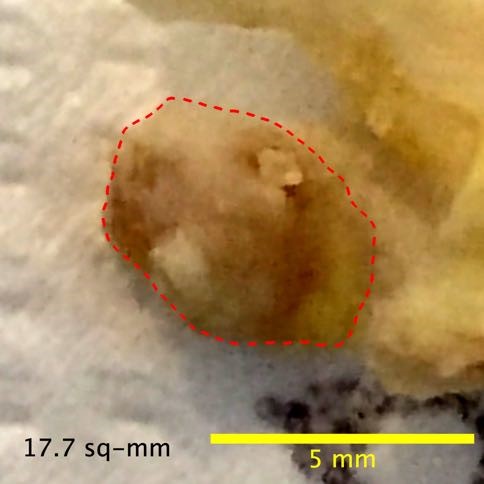


4

R


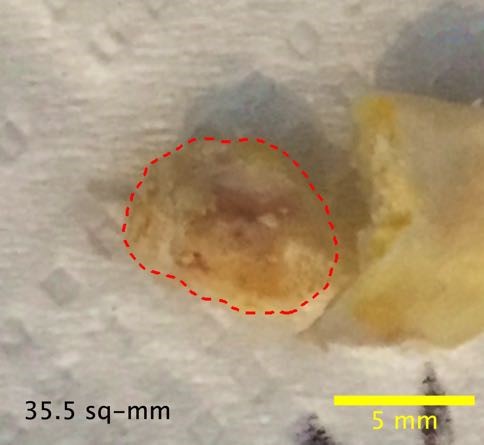


4

N

1


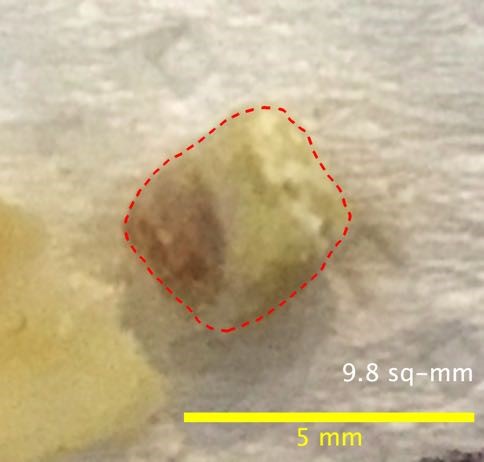


4

N

2


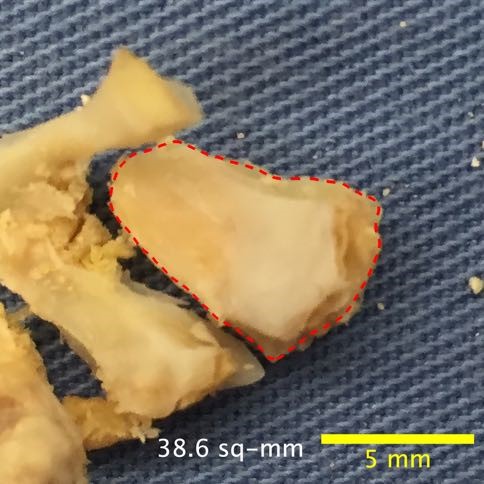


5

L

1


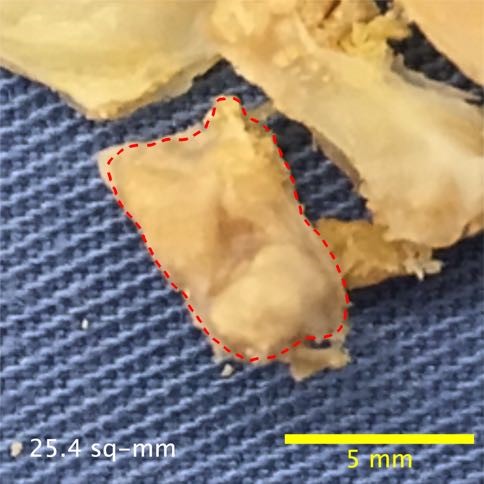


5

L

2


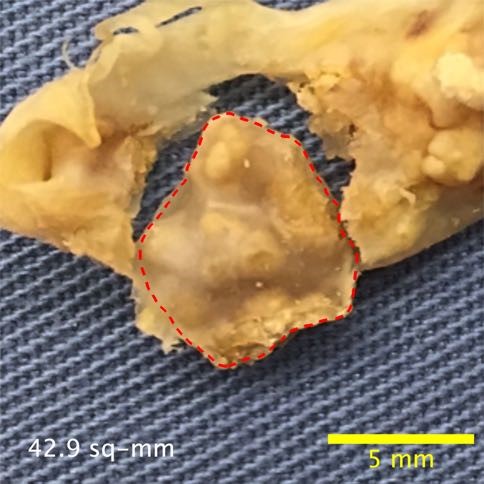


5

R


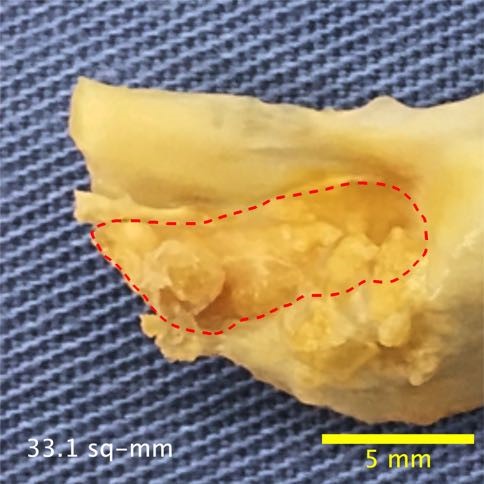


5

N


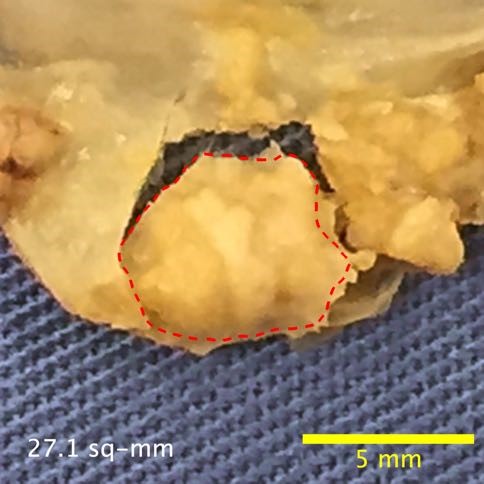


6

L


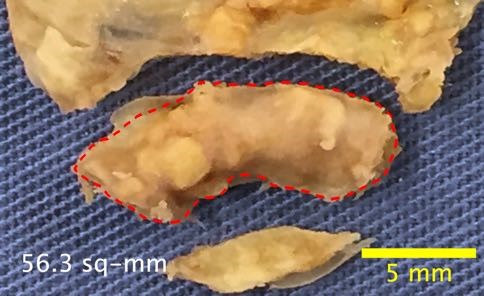


6

R


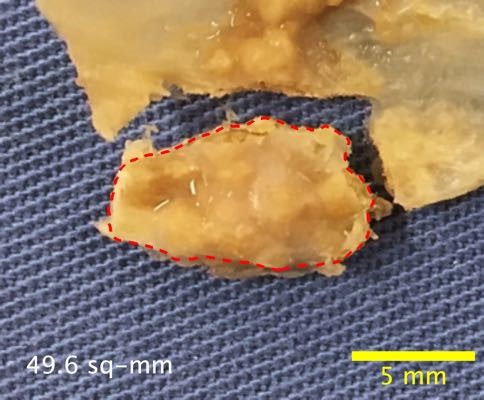


6

N

1


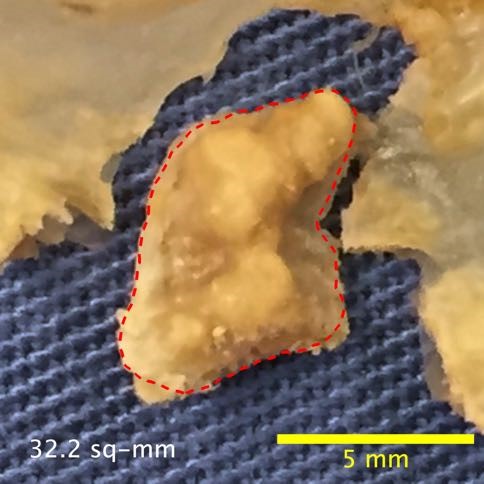


6

N

2


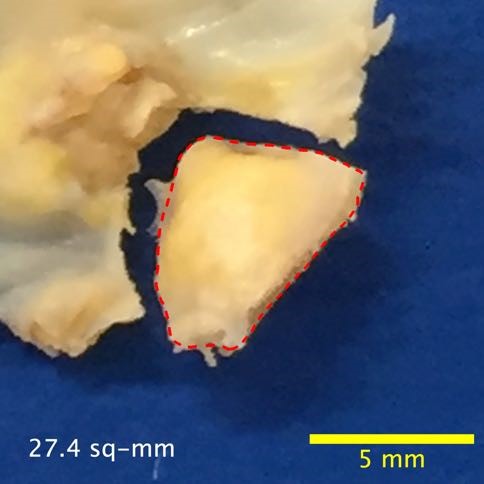


7

L


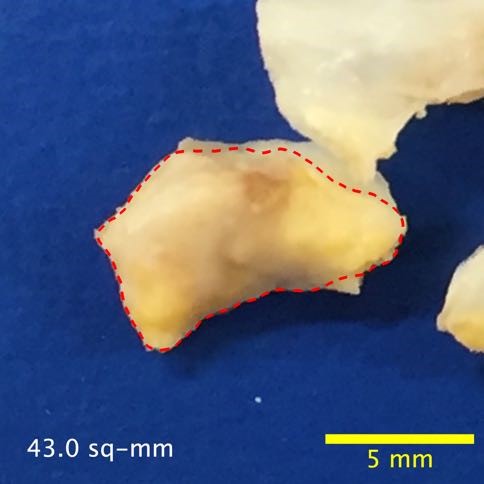


7

R

1


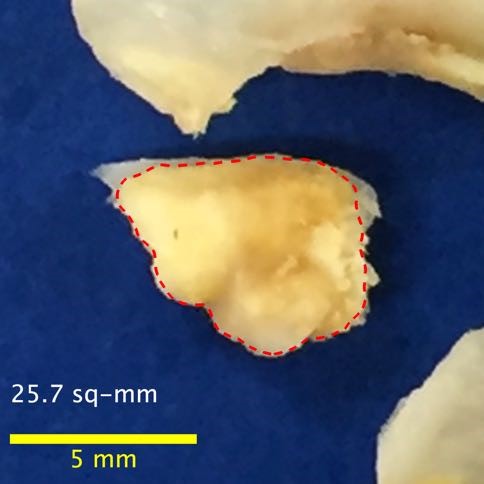


7

R

2


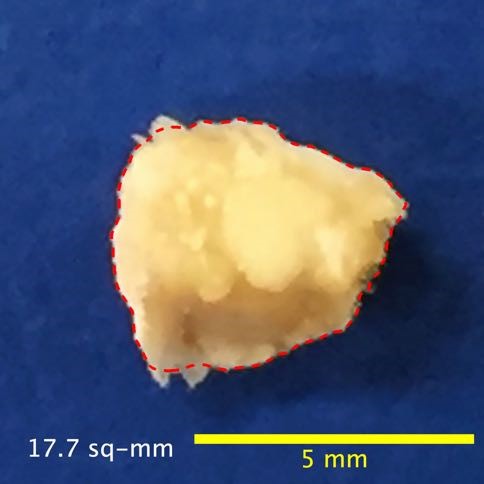


7

N

1


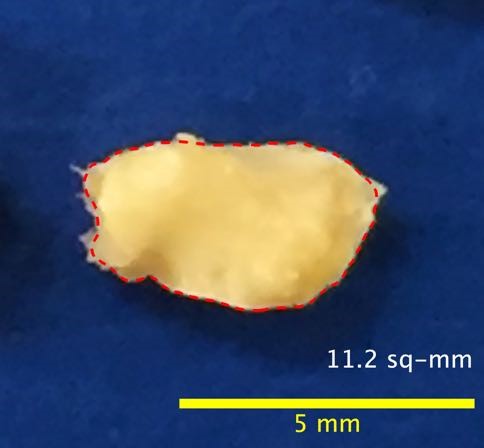


7

N

2


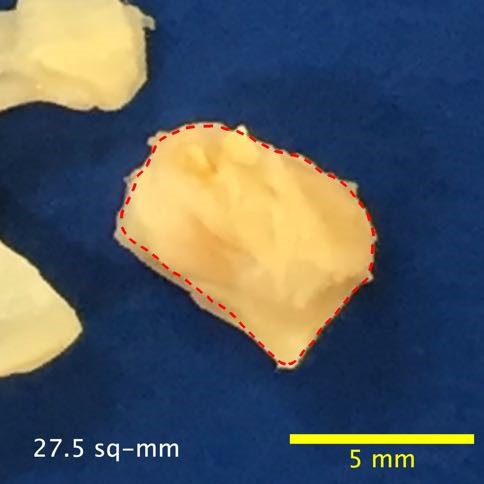


8

L


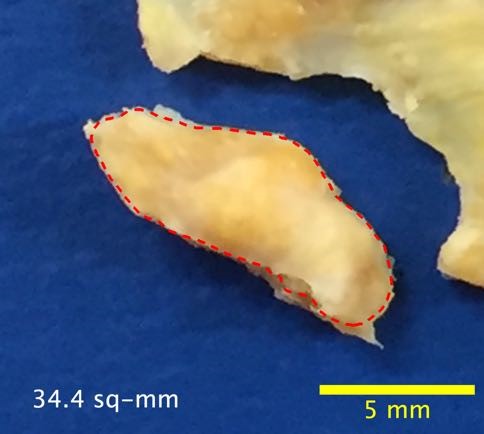


8

R


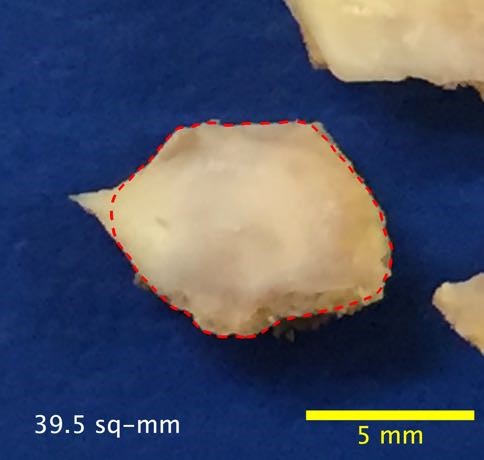


8

N


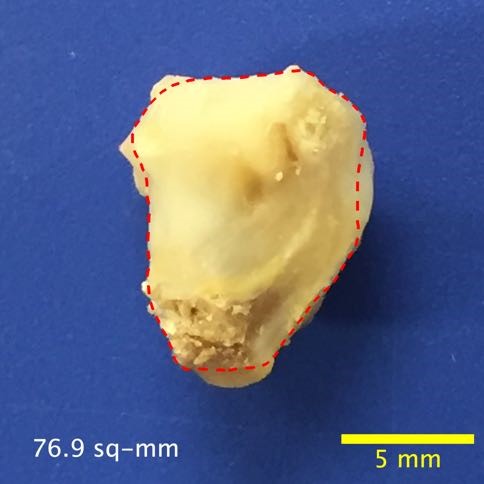


9

LR


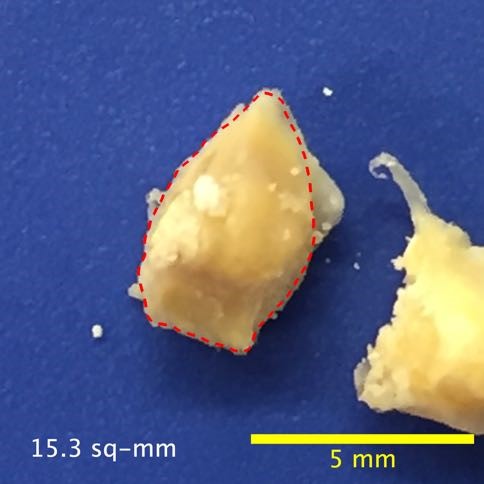


9

R

1


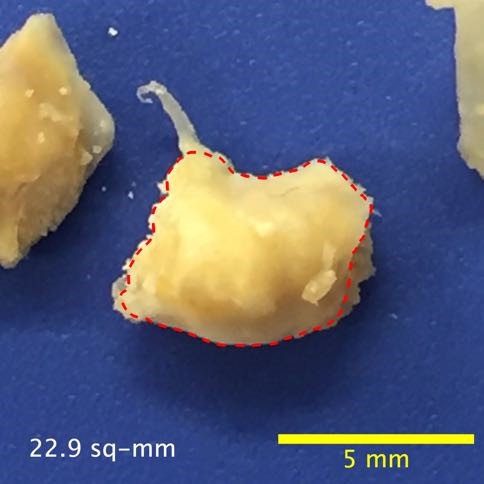


9

R

2


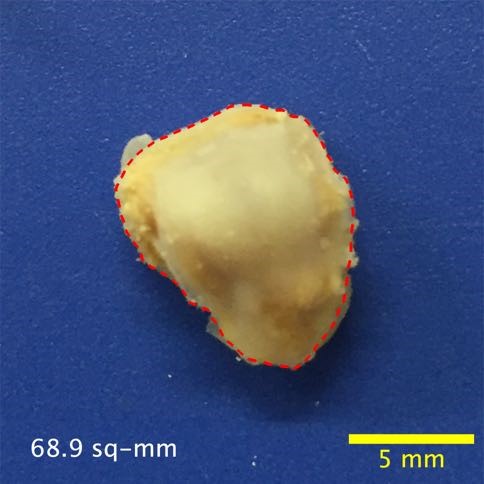


9

N

1


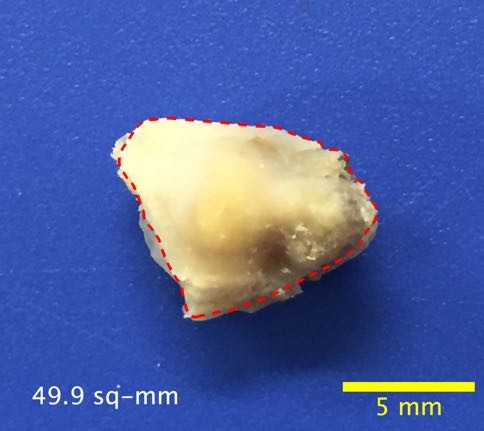


9

N

2


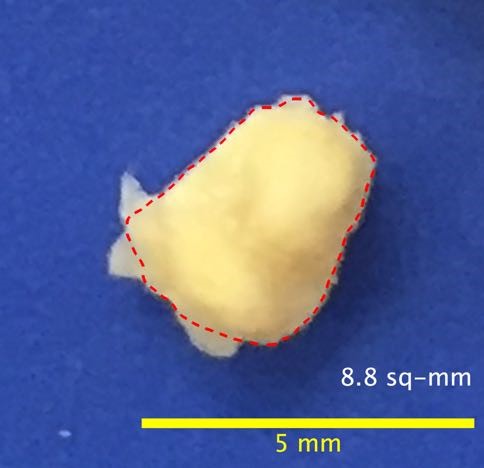


10

R


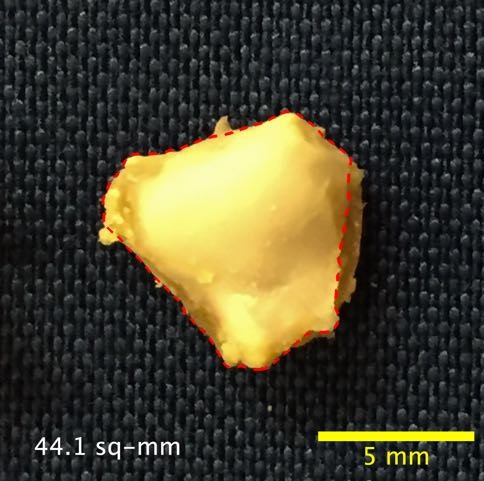


11

L

1


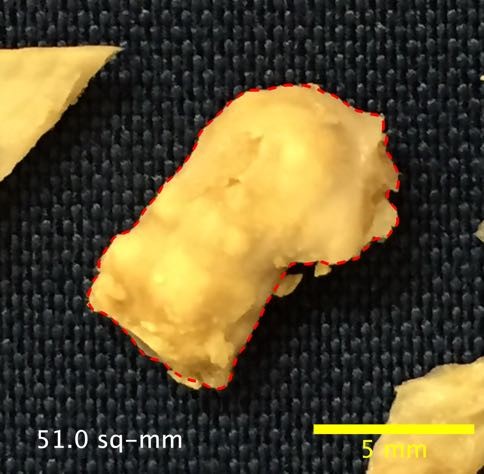


11

L

2


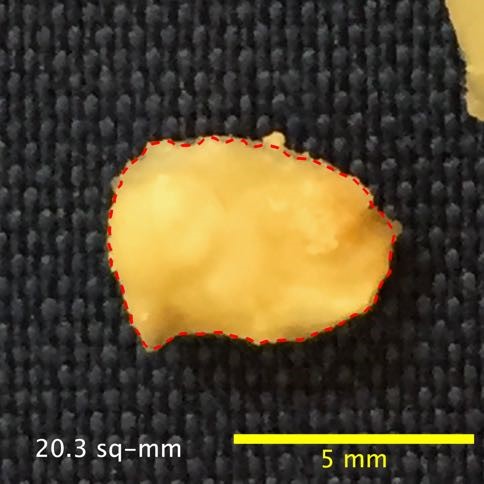


11

R


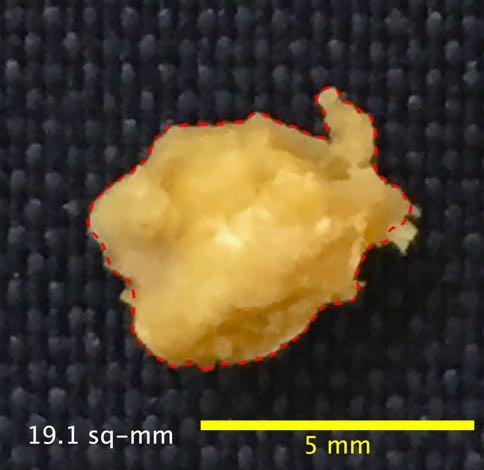


11

N

1


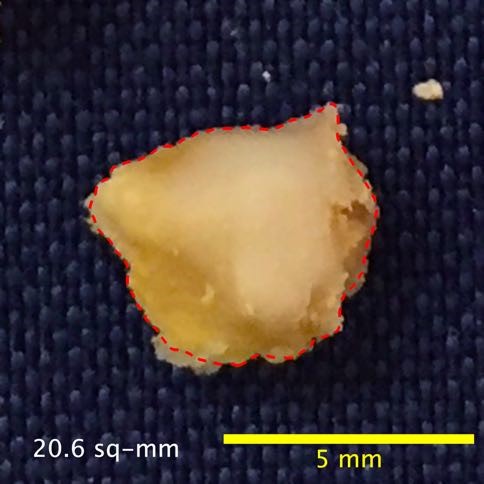


11

N

2


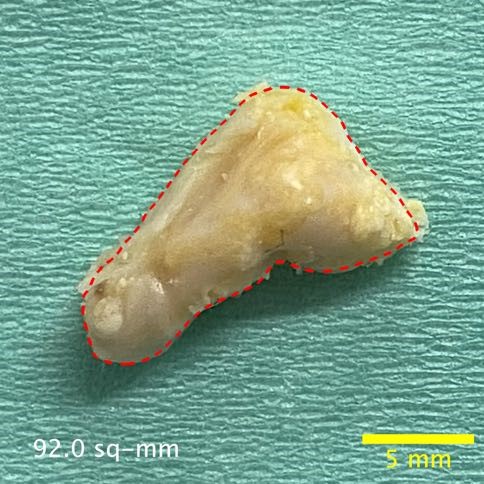


12

L


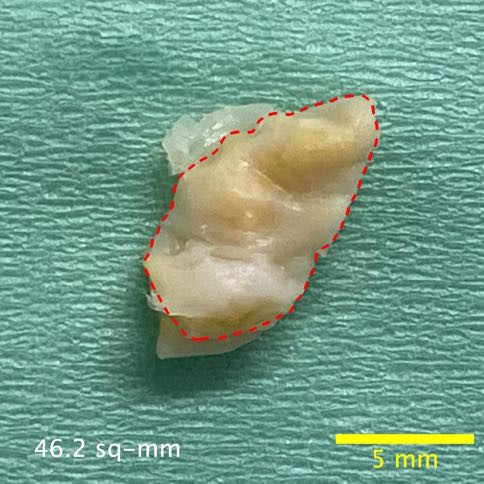


12

R


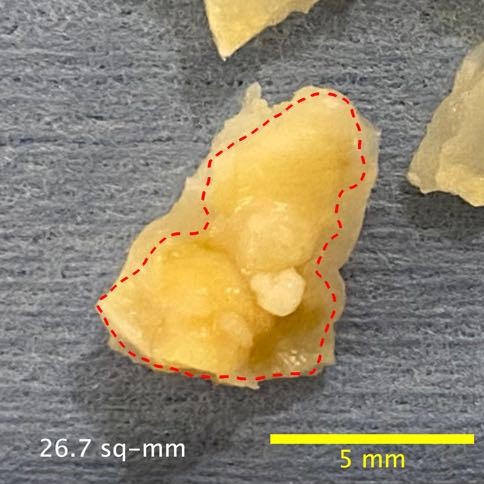


13

L


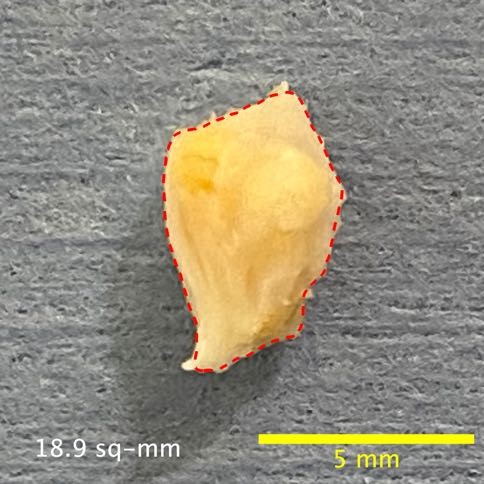


13

R


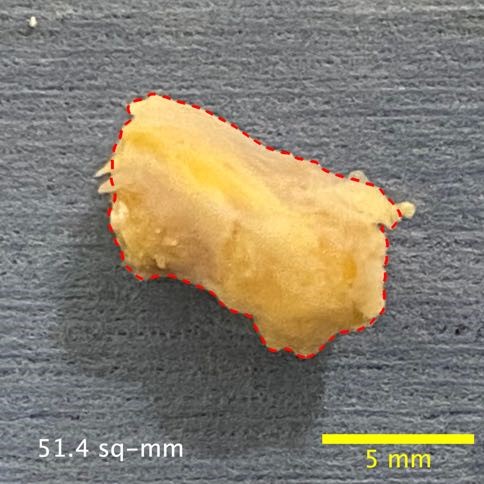


13

N

1


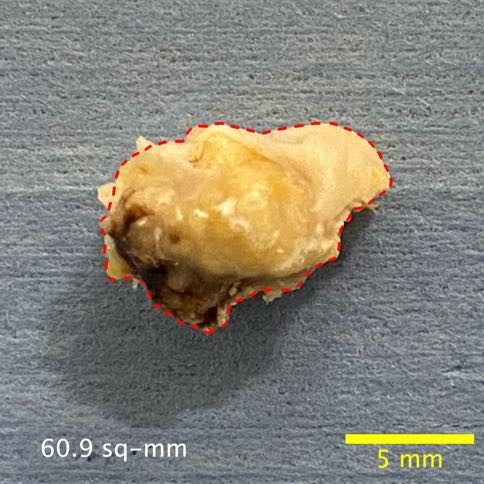


13

N

2


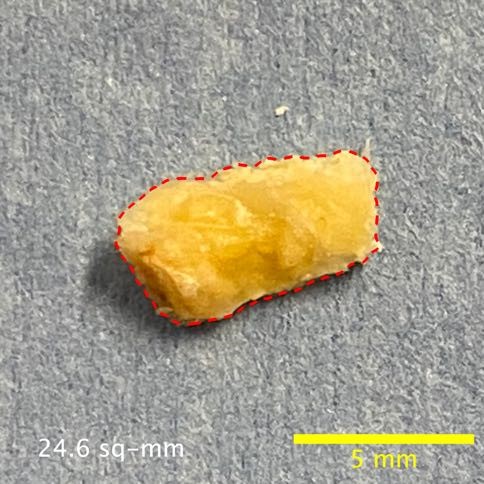


14

R

1


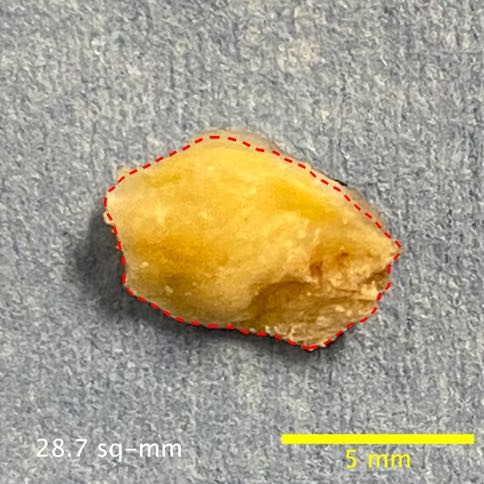


14

R

2


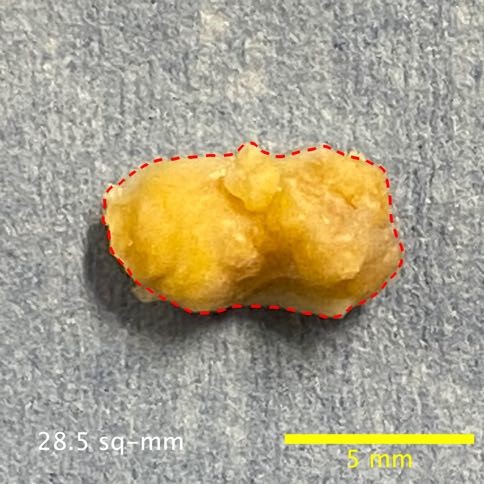


15

L

1


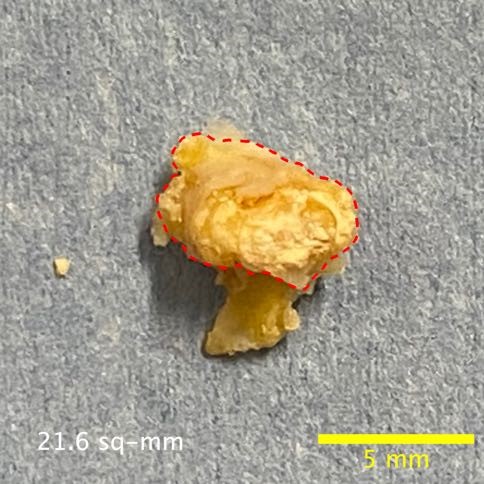


15

L

2


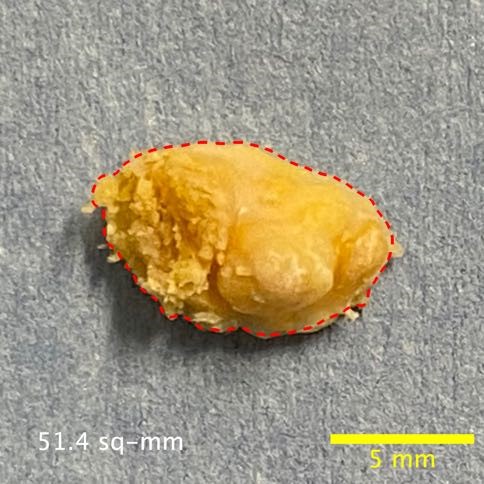


15

R

1


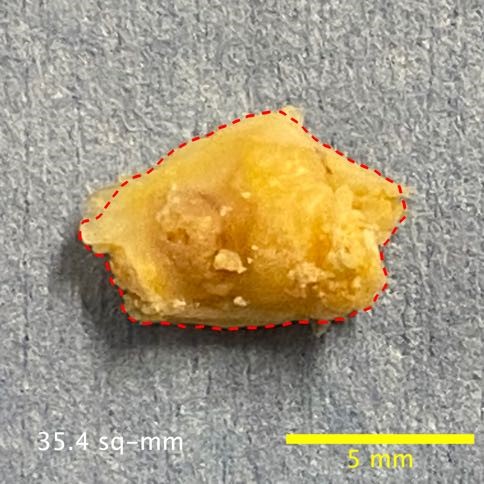


15

R

2


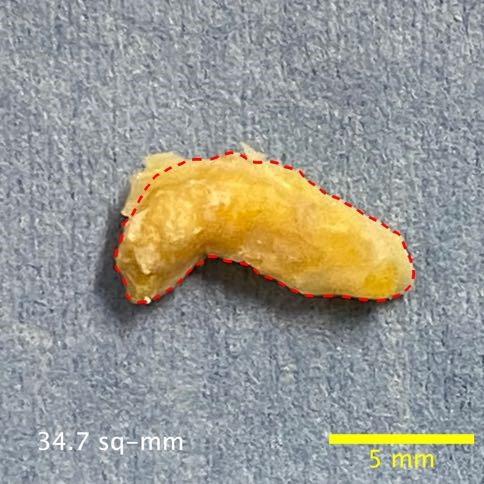


15

N


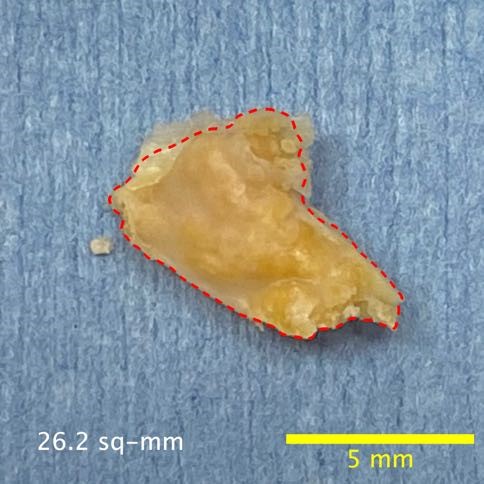


16

L


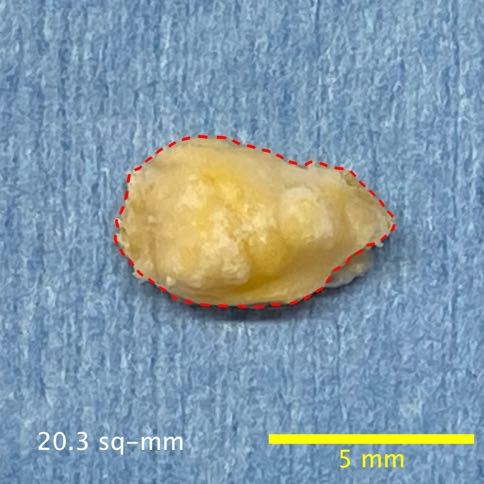


16

R

1


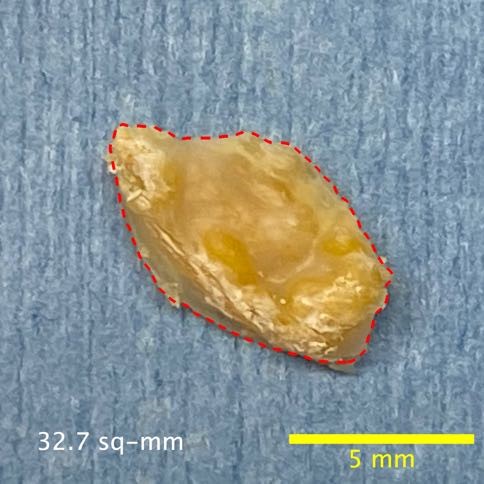


16

R

2


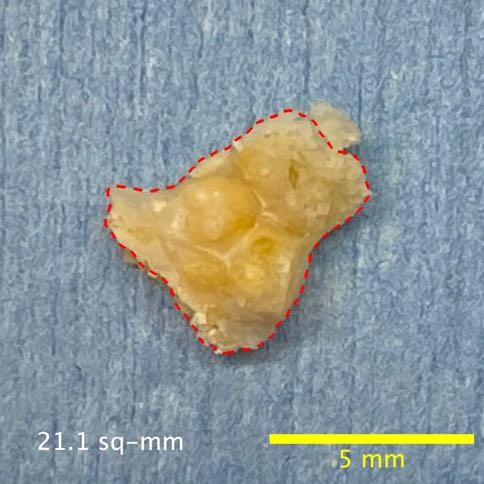


16

N


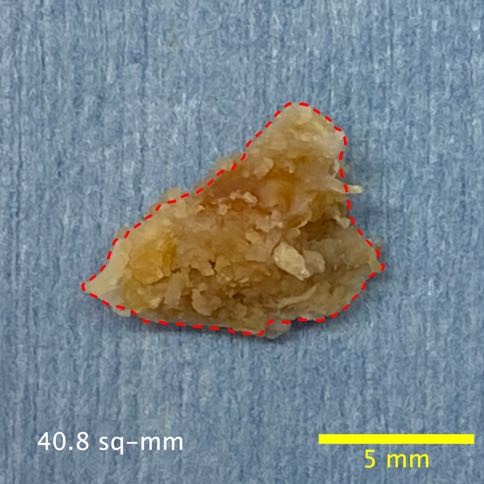


17

L


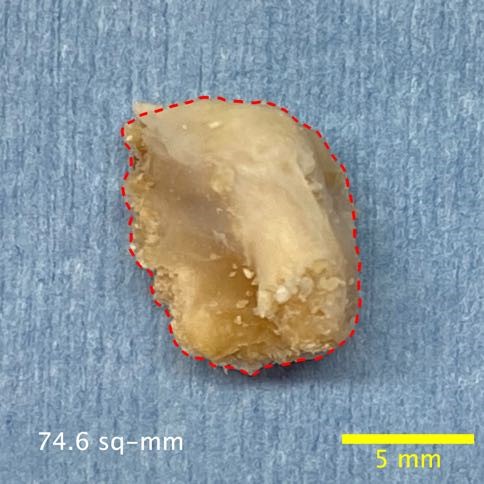


17

R


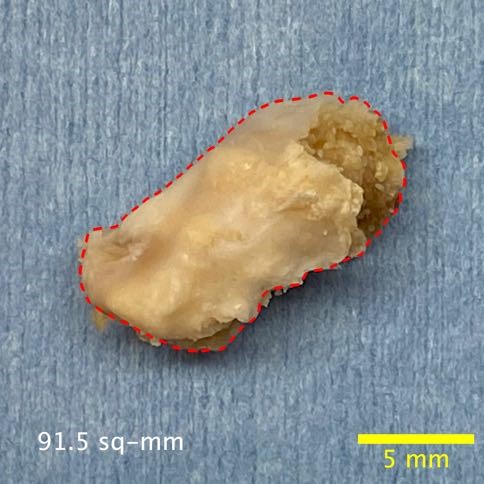


17

N


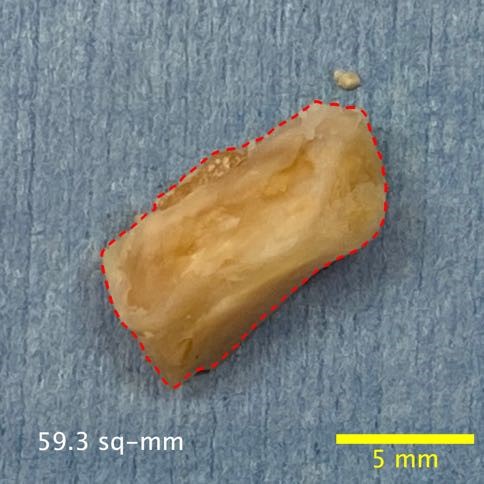


18

L


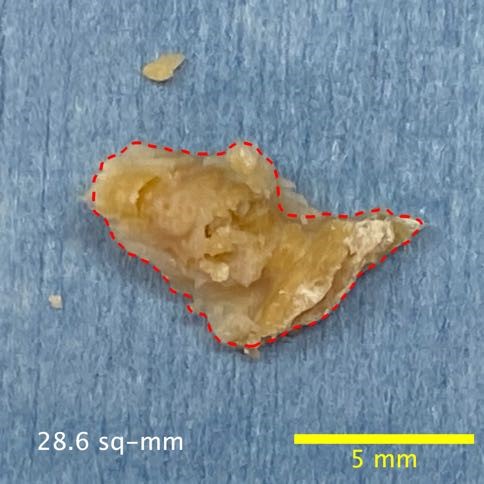


18

R


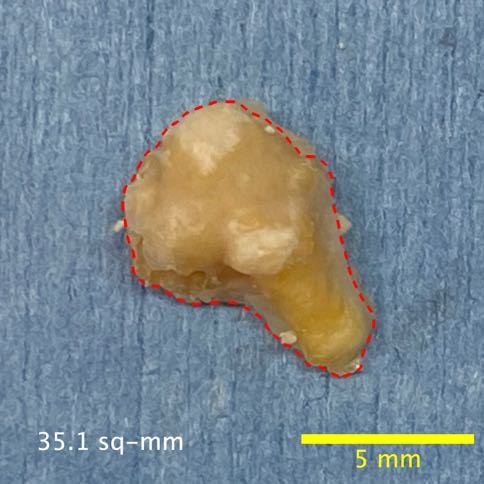


18

N


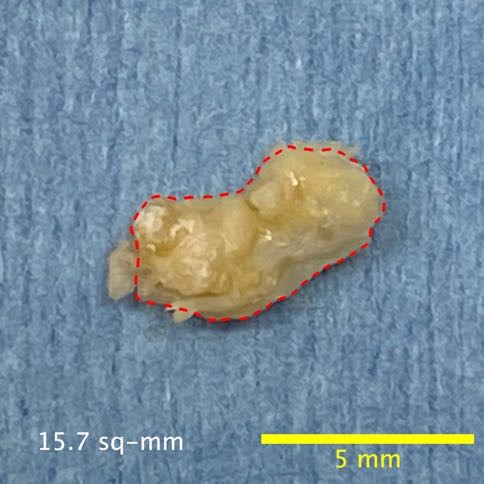


19

L


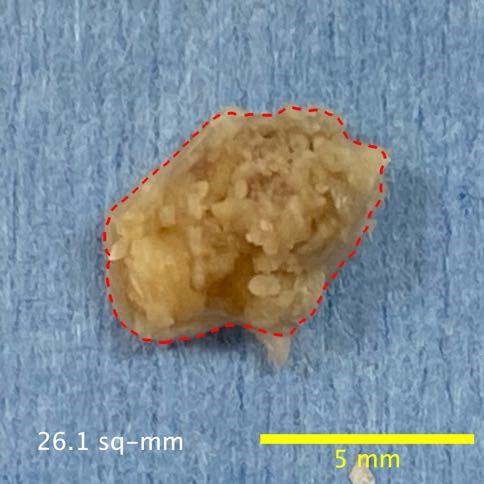


19

N

1


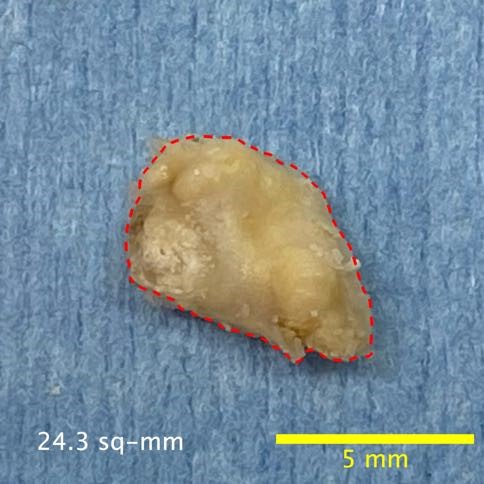


19

N

2


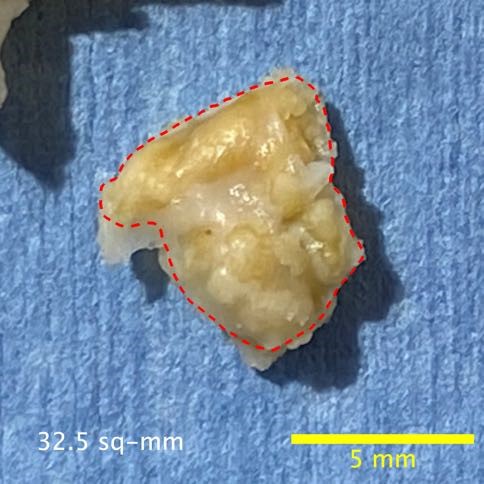


20

N


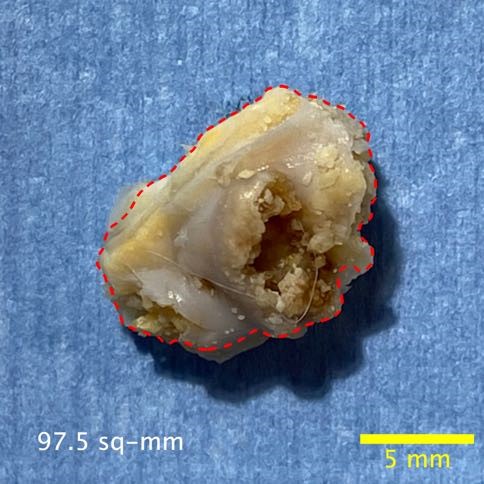


21

L


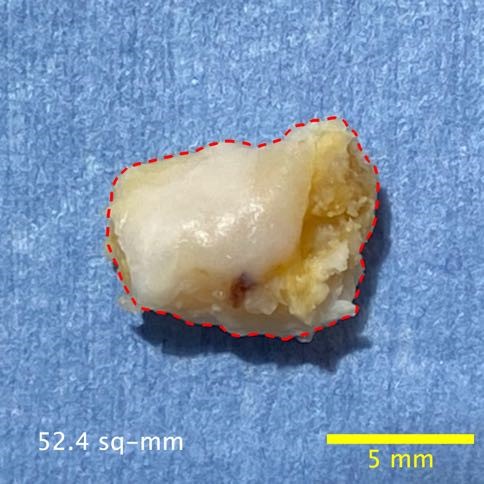


21

R


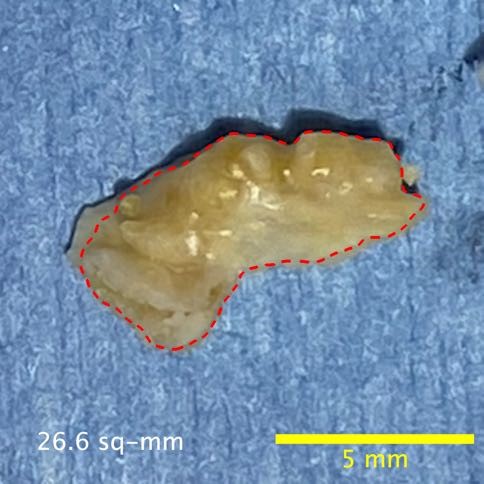


22

R

1


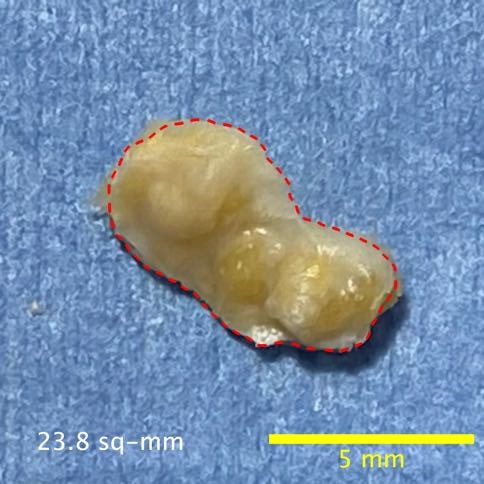


22

R

2


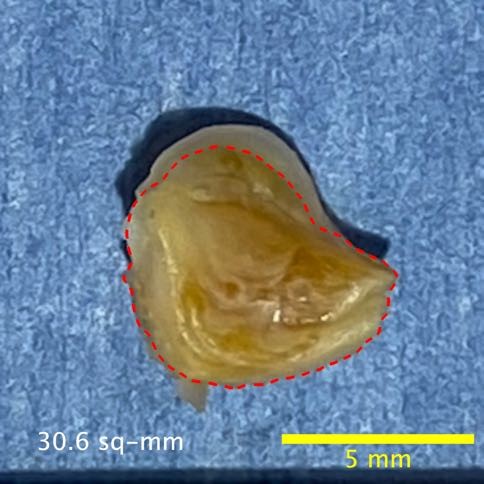


23

L


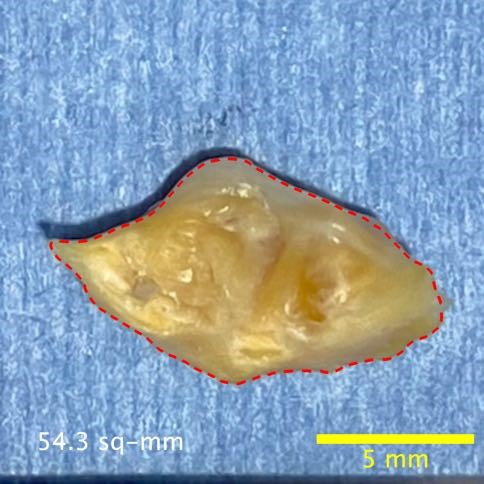


23

R


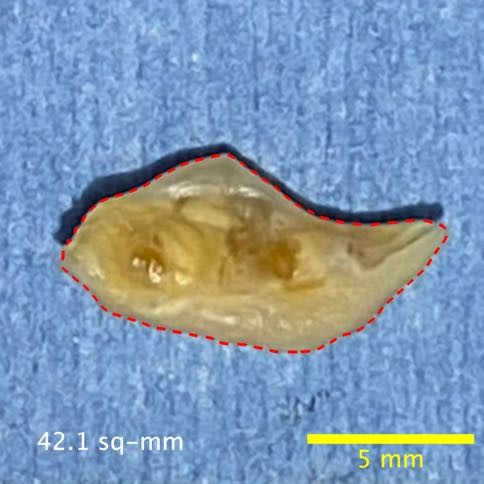


23

N


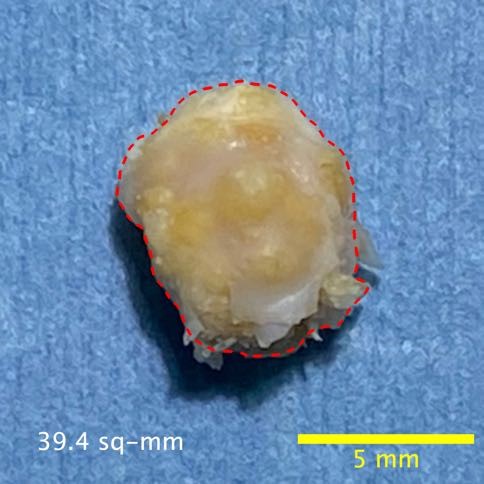


24

L


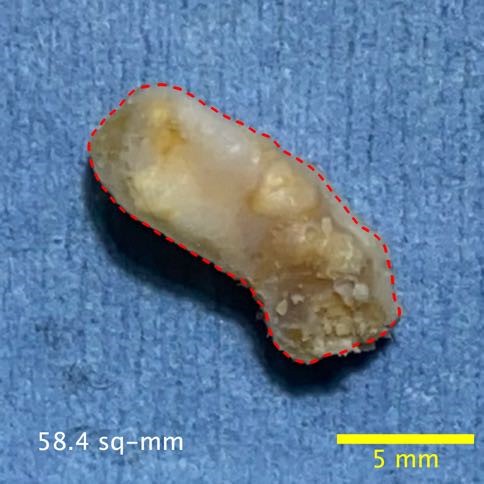


24

R


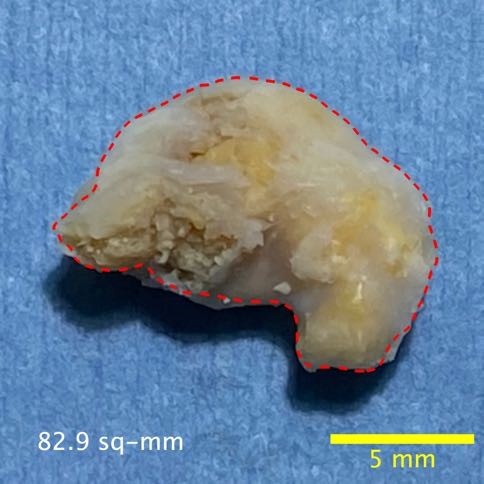


24

N


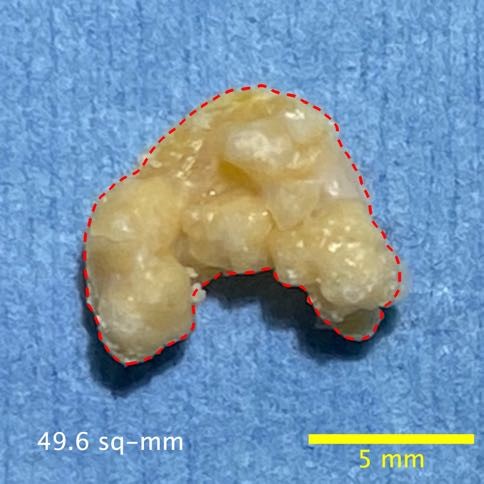


25

N


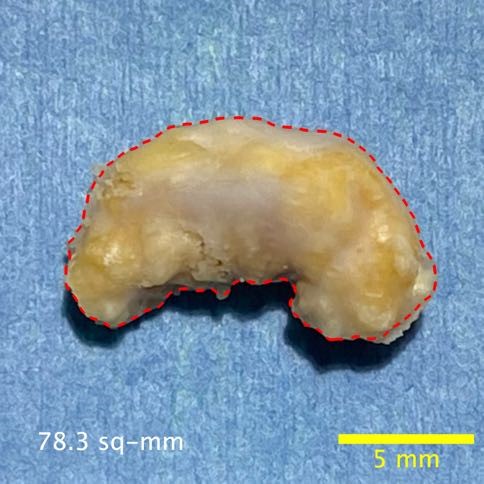


26

L


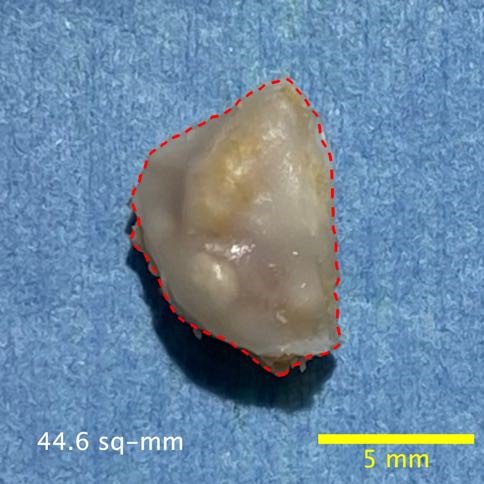


26

R


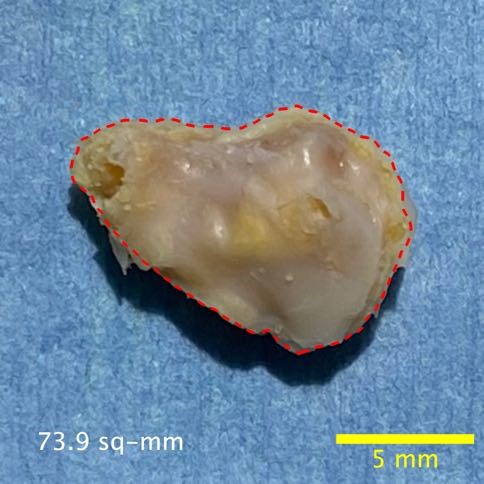


26

N


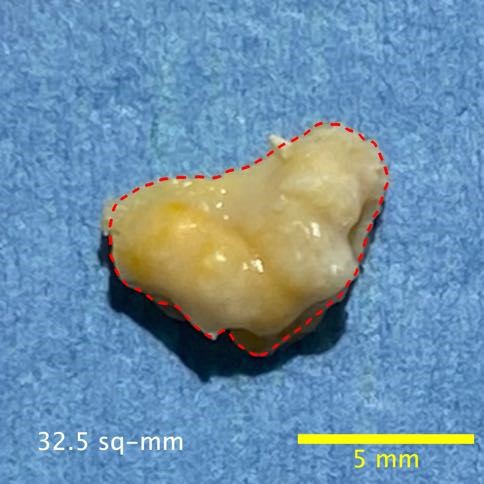


27

L

1


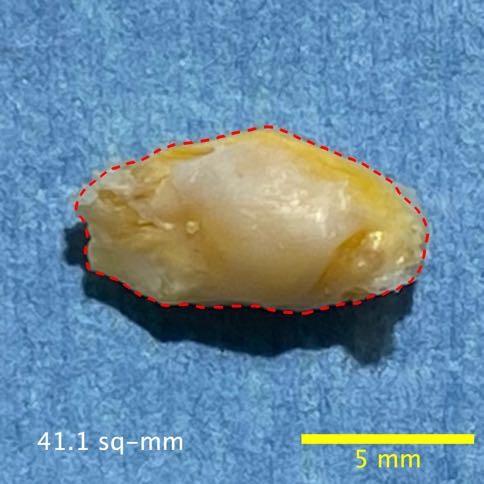


27

L

2


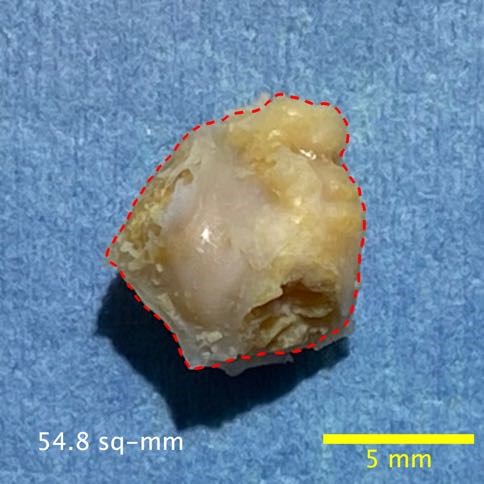


27

RN

1


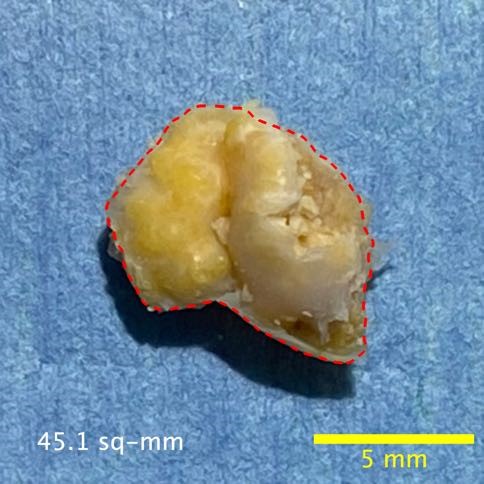


27

RN

2


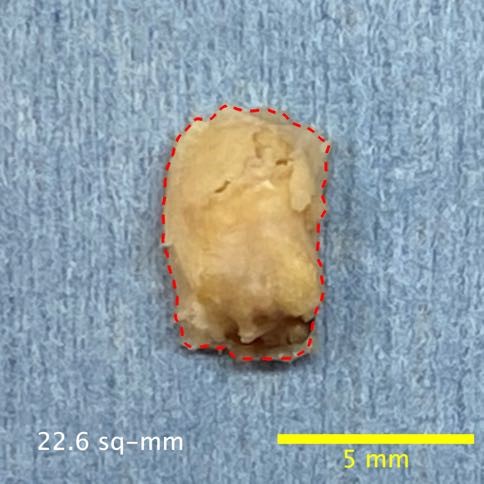


28

L

1


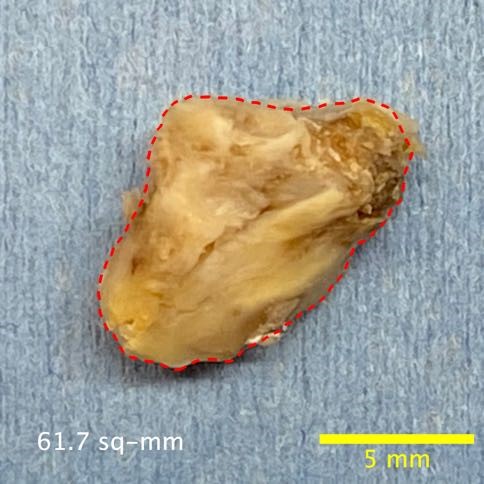


28

L

2


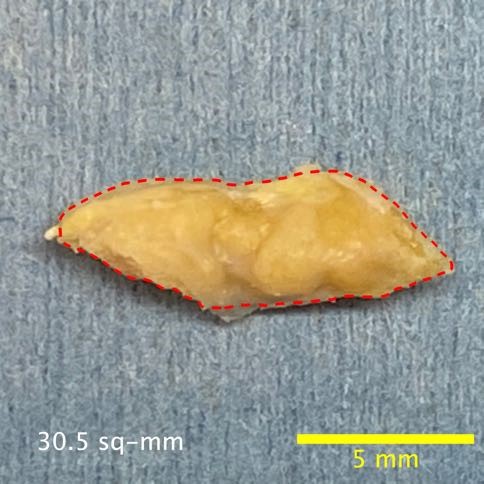


28

R


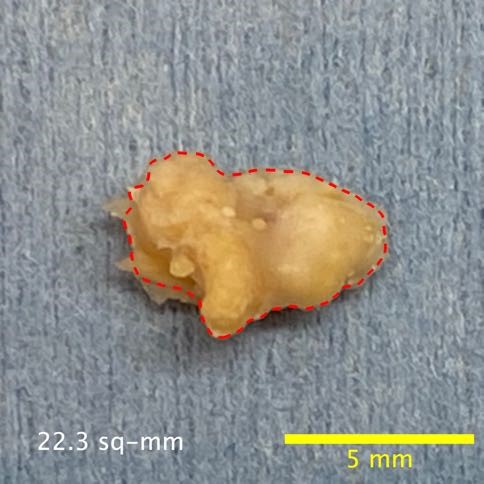


28

N


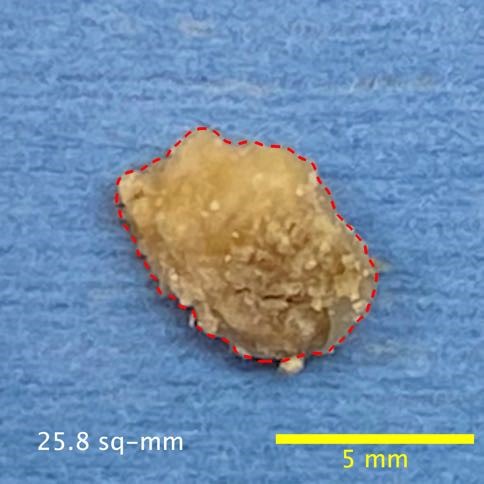


29

L

1


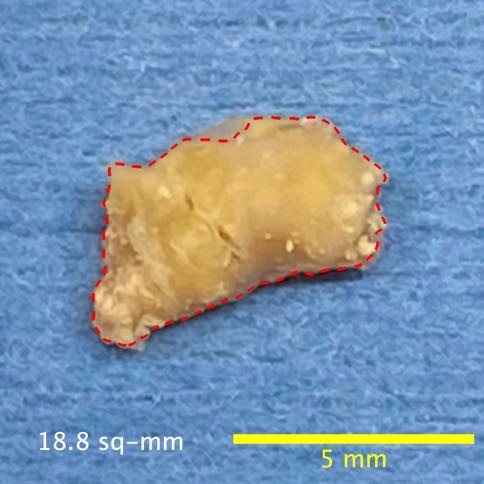


29

L

2


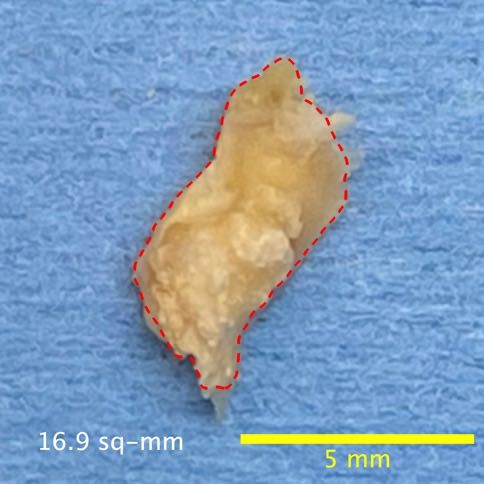


29

R

1


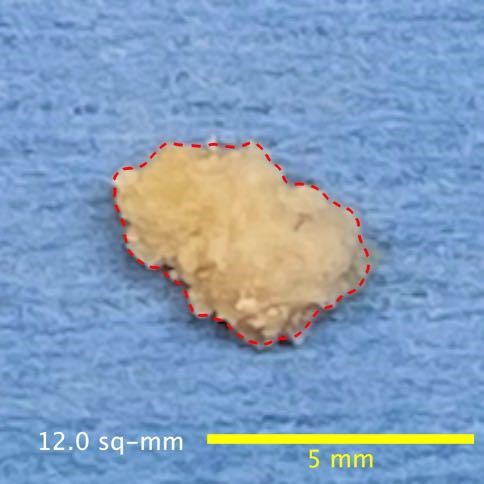


29

R

2


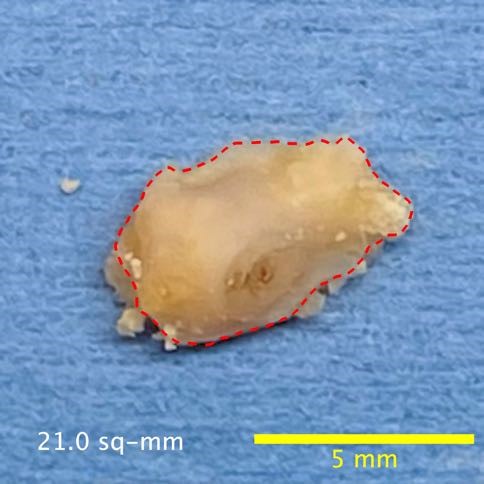


29

N

1


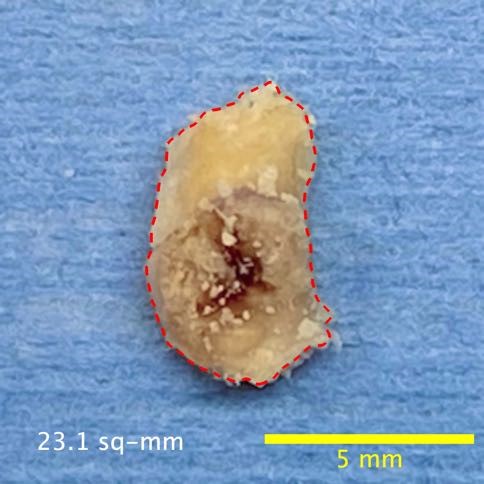


29

N

2


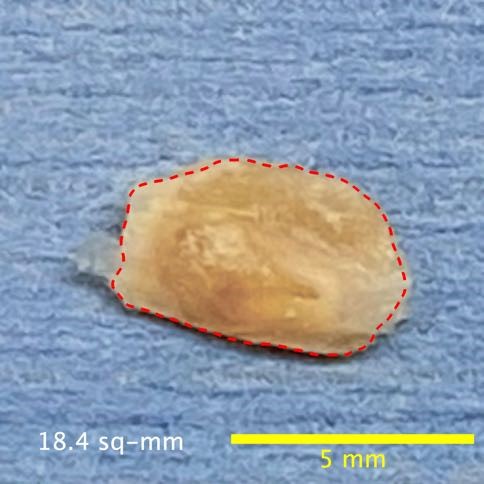


30

L


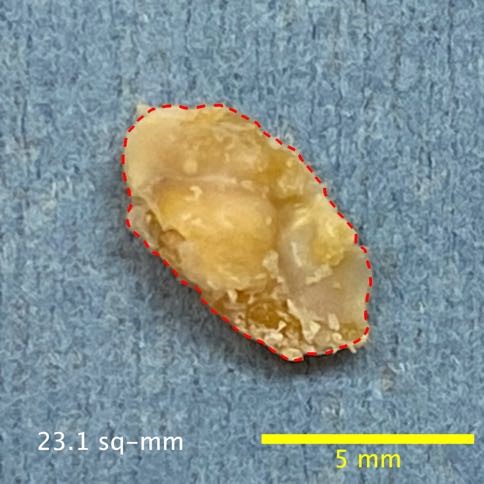


31

R


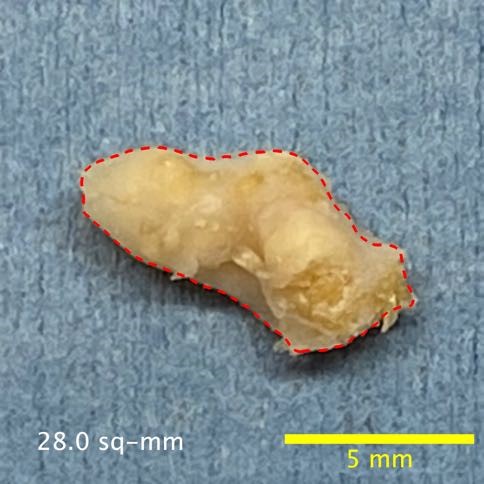


32

R

32

N

33

R

1

33

R

2

34

R

35

L

35

LR

35

R

35

N

36

R

36

N

37

L

37

R

38

R

38

N

39

LR

39

N

40

L

41

R

1

41

R

2

42

N

43

R

43

N

44

L

45

L

45

N

46

L

46

R

46

N

1

46

N

2

**Supplemental Figure 2.** Stress-strain curves of calcium nodules. The legend indicates the case number and cusp; for example, "1R" refers to the right coronary cusp of Case 1, matching those shown in Supplementary Figure 1. Each curve represents a centered moving average with a strain width of 0.05, applied to reduce high-frequency noise in the raw measurements. Compression Strength and Energy were calculated from these curves, as described in the Methods section.

1

L

1

R

1

N

2

L

-1

2

L

-2

2

R

2

N

3

L

3

R

3

N

-1

3

N

-2

4

L

4

R

4

N

-1

4

N

-2

5

L

-1

5

L

-2

5

R

5

N

6

L

6

R

6

N

-1

6

N

-2

7

L

7

R

-1

7

R

-2

7

N

-1

7

N

-2

8

L

8

R

8

N

9

LR

9

R

-1

9

R

-2

9

N

-1

9

N

-2

10

R

11

L

-1

11

L

-2

11

R

11

N

-1

11

N

-2

12

L

12

R

13

L

13

R

13

N

-1

13

N

-2

14

R

-1

14

R

-2

15

L

-1

15

L

-2

15

R

-1

15

R

-2

15

N

16

L

16

R

-1

16

R

-2

16

N

17

L

17

R

17

N

18

L

18

R

18

N

19

L

19

N

-1

19

N

-2

20

N

21

L

21

R

22

R

-1

22

R

-2

23

L

23

R

23

N

24

L

24

R

24

N

25

N

26

L

26

R

26

N

27

L

-1

27

L

-2

27

RN

-1

27

RN

-2

28

L

-1

28

L

-2

28

R

28

N

29

L

-1

29

L

-2

29

R

-1

29

R

-2

29

N

-1

29

N

-2

30

L

31

R

32

R

32

N

33

R

-1

33

R

-2

34

R

35

L

35

LR

35

R

35

N

36

R

36

N

37

L

37

R

38

R

38

N

39

LR

39

N

40

L

41

R

-1

41

R

-2

42

N

43

R

43

N

44

L

45

L

45

N

46

L

46

R

46

N

-1

46

N

-2
